# Selective depletion of tumor-associated SAMHD1 enhances chemotherapeutic efficacy and antitumor immune responses

**DOI:** 10.1038/s41392-025-02523-1

**Published:** 2025-12-15

**Authors:** Jing Sun, Wenwen Zheng, Zheng-Guo Zhang, Hongkun Zhou, Songdi Wang, Dingbo Huang, Xiao-Yi Hu, Qing-Feng Yu, Zhao-Xing Wu, Yi-Fei Shi, Runxin Ye, Fengyan Xia, Wangwei Li, Shurui Lyu, Yu Huang, Xu-Zhao Zhang, Fei Xu, Ke Zhao, Jie Yang, Juan Du, Jiaming Su, Yajuan Rui, Rongzhen Xu, Wei-Ming Yang, Cang Li, Jia ling Xu, Ruiyu Zhu, Xiaoguang Wang, Wei Wei, Xiao-Fang Yu

**Affiliations:** 1 Cancer Institute (Key Laboratory of Cancer Prevention and Intervention, China National Ministry of Education), The Second Affiliated Hospital, School of Medicine, Zhejiang University, Hangzhou, Zhejiang China; 2 Yale School of Medicine, Yale University, New Haven, CT USA; 3 Department of Hepatobiliary Surgery, Affiliated Hospital of Jiaxing University, Jiaxing, China; 4 Department of Hematology, the Second Affiliated Hospital, School of Medicine, Zhejiang University, Hangzhou, Zhejiang China; 5 School of Biological and Chemical Engineering, Zhejiang University of Science and Technology, Hangzhou, China; 6 Experimental Animal Research Center, Zhejiang Chinese Medical University, Hangzhou, China; 7 Institute of Virology and AIDS Research, First Hospital of Jilin University, Changchun, China; 8 Department of Molecular Microbiology and Immunology, Johns Hopkins University Bloomberg School of Public Health, Baltimore, MD USA; 9 College of Biosystems Engineering and Food Science, Zhejiang University, Hangzhou, China; 10 Cancer Center, The First Hospital of Jilin University, Changchun, Jilin China; 11 Institute of Translational Medicine, Key Laboratory of Organ Regeneration and Transplantation of Ministry of Education, The First Hospital of Jilin University, Changchun, Jilin China

**Keywords:** Haematological cancer, Drug development, Drug development, Molecular medicine

## Abstract

SAMHD1 is a human deoxyribonucleoside triphosphatase (dNTPase) known for its role as a restriction factor that targets a wide spectrum of viruses, its involvement in autoimmune disease Aicardi–Goutières syndrome (AGS), and its participation in innate immune regulation. The role of SAMHD1 in cancer, particularly its contribution to drug resistance, has gained increasing attention in recent years. One significant scientific challenge is how to inhibit SAMHD1 function in tumor cells while preserving its function in normal primary cells. Herein, we identified that increased SAMHD1 expression levels correlate with poor prognosis across multiple cancer types, and that SAMHD1 is upregulated in a variety of tumors. Through proteomic analysis and drug screening, we identified a promising strategy for selectively depleting tumor-associated SAMHD1 while minimizing its impact on SAMHD1 expression in key normal cell types. Our approach effectively enhanced tumor cytotoxicity when combined with multiple chemotherapeutic agents and suppressed tumor growth in vivo. Moreover, selective depletion of tumor-associated SAMHD1 activated innate immune responses, leading to enhanced tumor cell killing by immune cells. Collectively, these findings suggest that targeting tumor-specific SAMHD1 represents a novel and promising therapeutic strategy for cancers characterized by elevated SAMHD1 expression, offering potential for improved treatment outcomes in cancer patients with high SAMHD1 expression.

## Introduction

Sterile alpha motif and HD domain-containing protein 1 (SAMHD1) is a key deoxyribonucleoside triphosphatase (dNTPase)^1^ in humans, functioning primarily as a homo-tetramer.^2,3^ SAMHD1plays a pivotal role in regulating intracellular dNTP levels, which are essential for controlling cell proliferation and the cell cycle.^4^ This regulation is critical for maintaining genomic stability, particularly during DNA replication and repair processes. In myeloid cells and resting CD4 + T lymphocytes, SAMHD1 helps maintain low dNTPs levels, limiting viral DNA synthesis and restricting the infection of a variety of viruses, including HIV.^1,5–8^ Beyond its enzymatic dNTPase activity, SAMHD1 also exerts a dNTPase-independent viral restriction function,^9–12^ which further underscores its broad antiviral capabilities. Additionally, SAMHD1 plays an important role in inhibiting endogenous retroelements, such as LINE-1, ALU, and SVA.^13–15^ These retroelements, when left unchecked, can disrupt cellular functions and contribute to genomic instability, leading to tumorigenesis.

SAMHD1 mutations are linked to Aicardi-Goutieres syndrome (AGS), a severe genetic autoimmune encephalopathy.^16^ These mutations impair SAMHD1’s ability to restrict LINE-1, a long interspersed nuclear element that can trigger innate immune responses through the activation of the cGAS-STING and RIG-I-like receptor pathway.^17,18^ Moreover, SAMHD1 has been shown to interact with various immune signaling pathways, including MAVS and IKK complexes, which further enhances its role in immune modulation.^19,20^ Besides its antiviral function, SAMHD1 is also integral to maintaining cellular integrity by participating in DNA repair mechanisms, particularly in the repair of double-stranded breaks (DSBs),^21^ and in promoting the degradation of nascent DNA at stalled replication forks.^22^ Depletion of SAMHD1 results in the activation of innate immune activation, including the production of type-I interferon and inflammatory factors.^23,24^

Acute myeloid leukemia (AML) is a common and aggressive form of hematological malignancy in adults, defined by rapid expansion of abnormal cells within the myeloid lineage. Despite progress in therapeutic strategies, AML is still characterized by a poor prognosis, and the 5-year survival rate for adult patients stands at less than 30%.^25–27^ The standard first-line therapy for AML involves cytarabine (ara-C) and anthracycline, two chemotherapy agents that are effective in the initial treatment phase.^28–30^ However, resistance to ara-C remains the primary cause of treatment failure, particularly in patients with relapsed AML.^31^ SAMHD1 has been implicated in this resistance, as it plays a crucial role in regulating dNTP levels within the cell, which in turn affects the efficacy of nucleoside-based chemotherapies like ara-C.^32,33^ SAMHD1’s role as a dNTP triphosphohydrolase is central to its ability to limit the availability of nucleotides for DNA synthesis, thus contributing to resistance against chemotherapeutic drugs that rely on nucleotide incorporation. Beyond ara-C, nucleotide analogs serve as common therapeutic agents for a range of malignancies, and SAMHD1-mediated resistance to these drugs presents a significant challenge for overcoming drug resistant. To address this issue, several strategies have been explored to inhibit tumor-associated SAMHD1, particularly in the context of AML For instance, the HIV-2 Vpx protein, which can bind to SAMHD1, induces polyubiquitination and subsequent proteasomal degradation of the protein. Studies have demonstrated that introducing Vpx into AML cells has been shown to deplete SAMHD1, thereby enhancing the anti-tumor effects of ara-C.^1,22^ However, while promising, applying this approach in vivo presents significant challenges, such as achieving selective targeting of SAMHD1 in tumor cells without affecting normal cells. Another potential strategy involves the development of small molecule inhibitors of SAMHD1, although these inhibitors face difficulties in selectively targeting SAMHD1 in tumor cells while sparing normal cells, which remain critical for immune function and viral defense.

Selectively depletion of SAMHD1 in tumors holds the potential to enhance the efficacy of various anti-cancer therapies and promotes innate immune activation, which in turn benefits the host’s anti-tumor immune responses. In this study, we developed a strategy to selectively target and deplete tumor-specific SAMHD1 while minimizing its impact on normal cells. This approach increased the cytotoxicity of several chemotherapy agents and inhibited tumor cell growth both in vitro and in vivo. Furthermore, depletion of tumor-associated SAMHD1 triggered innate immune responses and improved tumor cell killing by natural killer (NK) cells. These findings suggest that targeting SAMHD1 could not only improve the response to chemotherapy but also contribute to the activation of the host’s immune system, potentially providing a dual mechanism of action in cancer treatment. Moreover, SAMHD1 expression is upregulated in many types of tumors, including AML, and elevated levels are associated with poor prognosis in patients. Thus, our strategy of selectively depleting of tumor-associated SAMHD1 presents a promising therapeutic approach, especially for cancers characterized by high SAMHD1 expression.

## Results

### Identification of drugs depleting tumor-associated SAMHD1

**Fig. 1:**
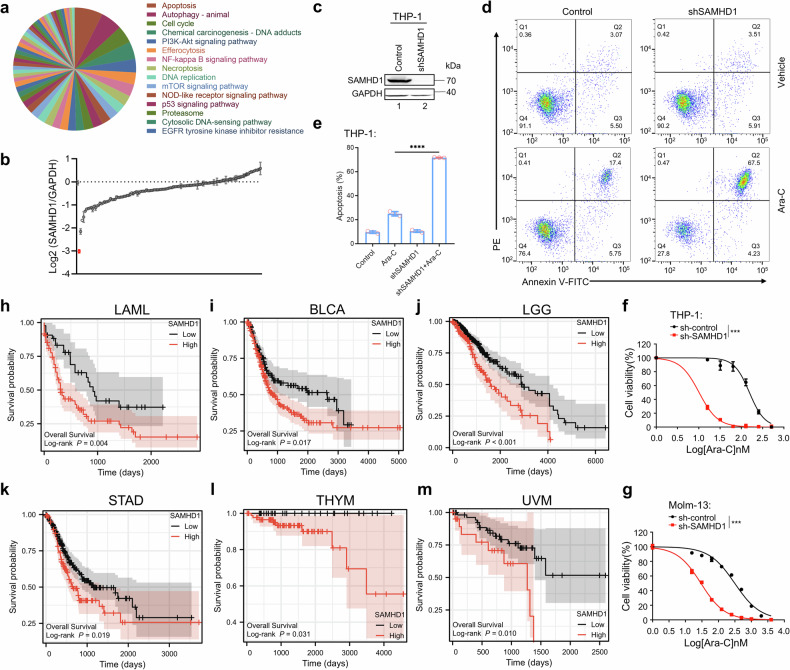
Identification of drugs depleting tumor-associated SAMHD1. **a**, **b** To identify small-molecule drugs capable of effectively downregulating SAMHD1 protein expression, a functional screen of 122 small-molecule drugs covering 121 cellular signaling pathways was conducted. Western blot analysis was used to evaluate their regulatory effects on SAMHD1 protein expression. **a** Pie chart showing the distribution of the 121 cellular pathways targeted by the 122 screened drugs. Different colors represent distinct pathway categories, with the proportional share of each color indicating the percentage of drugs corresponding to each pathway category relative to the total. The top 15 most represented pathways (by drug count) are annotated individually. **b** The effects of drugs on SAMHD1 expression were examined by western blot and quantified using ImageJ. STA-9090, highlighted in red, was found to be the most potent in degrading SAMHD1 protein expression level among the 122 drugs (*n* = 3). **c** SAMHD1 in THP-1 cells was knocked down via shRNA transduction. The expression level of endogenous SAMHD1 in the cells was verified by Western blot, with GAPDH used as the internal reference protein. **d**, **e** SAMHD1-knockdown or control THP-1 cells were treated with 100 nM cytarabine for 24 h, and cell apoptosis rate was detected by flow cytometry (*n* = 3). **f**, **g** Cytotoxicity assay of cytarabine in SAMHD1 knockdown **f** THP-1 and **g** Molm-13 cells (*n* = 3). **h**–**m** Kaplan–Meier plots of various cancer patients after inquartation for SAMHD1 mRNA expression levels. Hazard ratio (HR) and *p* values are indicated. LAML acute myeloid leukemia, BRCA breast invasive carcinoma, LGG brain lower-grade glioma, STAD stomach adenocarcinoma, THYM thymoma, UVM uveal melanoma

As SAMHD1 mediates the poor response of cancer patients to chemotherapeutic treatment,^32,33^ depletion of SAMHD1 in tumor cells may offer a new anticancer strategy. Thus, we screened FDA-approved small-molecule drugs covering multiple cellular signaling pathways for their abilities to reduce tumor-associated SAMHD1. The effects of drugs on SAMHD1 expression were examined by western blot followed by quantification using ImageJ (Fig. 1a and Table S1). As shown in Fig. 1b, STA-9090 was the most effective drug for depleting SAMHD1 in the tumor cell line THP-1. Knocking down SAMHD1 significantly promoted ara-C-triggered cell apoptosis (Fig. 5c–e), which is consistent with previous reports.^34^ The promotion of ara-C cytotoxicity when SAMHD1 expression was silenced in THP-1 and Molm-13 cells was simultaneously verified (Fig. 1f, g). Upon analyzing the profiles of patients with different cancer types in TCGA (The Cancer Genome Atlas) database, as shown in Fig. 1h–m, we found that higher SAMHD1 expression could be linked to worse patient survival in AML, breast invasive carcinoma, brain lower-grade glioma, stomach adenocarcinoma, thymoma, and uveal melanoma. We found HSP90 expression had variable influences on SAMHD1 expression and tumor patient’s survival (Supplementary Fig. 1). Therefore, reducing SAMHD1 expression in tumor cells may offer new antitumor strategies.

**Fig. 2:**
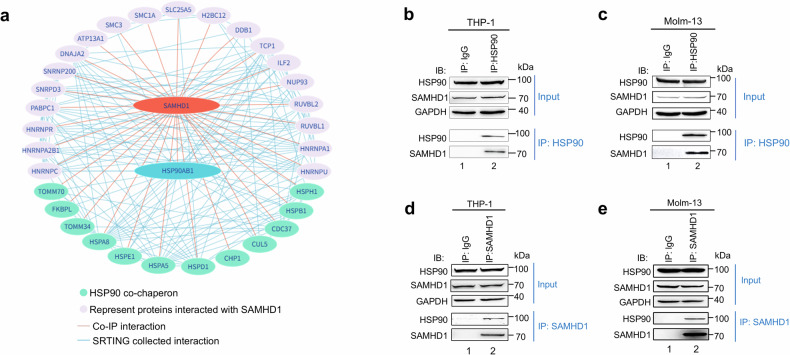
Characterization of the binding between SAMHD1 and HSP90. **a** Identification of HSP90 as a novel SAMHD1-binding protein. HEK293T cells were transfected with SAMHD1-HA expression vector or empty control vector. Cell lysates were prepared 36 h post-transfection, and HA-tagged SAMHD1 was immunoprecipitated using HA-immobilized agarose beads. The samples were then subject to MS analysis. HSP90 was found to be one of the proteins bindings to SAMHD1. **b**–**e** Confirmation of endogenous SAMHD1-HSP90 interaction in AML-derived cells. The endogenous HSP90-SAMHD1 interaction was analyzed by co-IP assays using anti-HSP90 or control IgG antibody with samples from **b** THP-1 and **c** Molm-13. The endogenous SAMHD1-HSP90 interaction was analyzed by co-IP assays using anti-SAMHD1 or control IgG antibody with samples from **d** THP-1 and **e** Molm-13 (*n* = 3)

To investigate the potential regulation mechanisms of SAMHD1, we performed SAMHD1 pull-down experiments, followed by mass spectrometric (MS) analysis to characterize its cofactors as previously described.^35^ Among the proteins coprecipitated with SAMHD1 (Fig. 2a; Table S1), HSP90 is particularly interesting because many client proteins are recognized to be modulated by HSP90 chaperones.^36^ We noticed that many known members of HSP90 complexes also interacted with SAMHD1 (Fig. 2a, blue color). SAMHD1 has been shown to influence viral replication.^24,37^ Factors interacting with SAMHD1 such as DDB1 and hnRNP A1, have been linked to viral infection including HIV-1^1,6,38^ and SARS-COV-2.^39^ We first confirmed the interaction between SAMHD1 and HSP90 in HEK293T cells by co-IP and western blot analysis (Supplementary Fig. 2a). We also found that endogenous SAMHD1 coimmunoprecipitated with HSP90 in cells from two AML cell lines, namely, THP-1 (Fig. 2b) and Molm-13 cells (Fig. 2c). We confirmed by reciprocal experiments that endogenous HSP90 coimmunoprecipitated with SAMHD1 in the THP-1 (Fig. 1d) and Molm-13 (Fig. 1e) cell lines. In addition, we observed that SAMHD1 and HSP90 colocalized in Molm-13 cells (Supplementary Fig. 2b), as revealed by immunofluorescent staining, identifying SAMHD1 as a novel HSP90 client protein. Moreover, our data demonstrated that SAMHD1 amino acids 344-626 play an important role in mediating HSP90 interaction (Supplementary Fig. 2c). We found that the NTD, LR, and CTD domains of HSP90 all contributed to its interaction with SAMHD1, while the ATPase activity of HSP90 (T110I mutation)^40^ is not required for this binding (Supplementary Fig. 2d, e). Our data were consistent with previous reports regarding HSP90 and Tau interaction.^41^

### Selective depletion of SAMHD1 in multiple tumor cell types, but not in normal cells

**Fig. 3:**
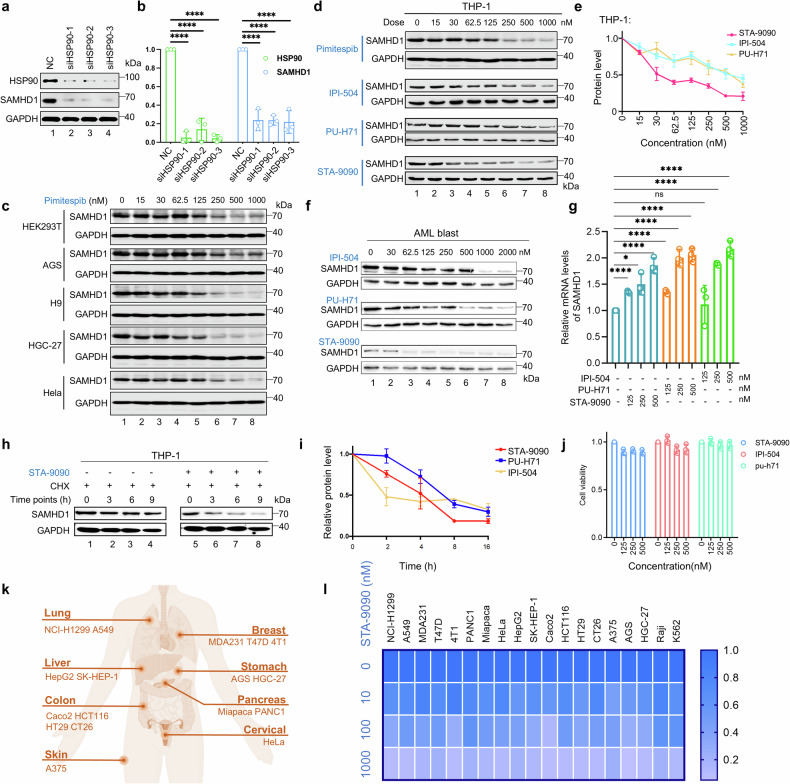
Selective and efficient depletion of SAMHD1 in various tumor types following HSP90 inhibition. **a** siRNA-mediated knockdown of HSP90 in Molm-13 cells reduced SAMHD1 protein abundance (*n* = 3). **b** Quantification of SAMHD1 and HSP90 protein levels, normalized to GAPDH; Data are presented as mean ± s.d. (*p < 0.05; **p < 0.01; ***p < 0.001; ****p < 0.0001). **c** HEK 293T, AGS, H9, HGC-27, HeLa and **d** Inoculation of THP-1 cells into 12-well cell culture plates and treated with indicated doses of Pimitespib, IPI-504, PU-H71, or STA-9090 for 18 h. SAMHD1 protein levels were analyzed by western blot (*n* = 3). **e** Protein quantification results from the experiments in (**d**) (*n* = 3). **f** Western blot assay of SAMHD1 protein levels in primary AML blasts derived from patients, following exposure to different concentrations of HSP90 inhibitors for 18 h (*n* = 2 patient samples). **g** RT-qPCR analysis of SAMHD1 mRNA levels in THP-1 cells treated with indicated concentrations of HSP90 inhibitors (IPI-504, PU-H71, or STA-9090) for 18 h (*n* = 3), with GAPDH as a normalization control. Data are presented as mean ± s.d. **h** Cycloheximide (CHX) chase analysis of SAMHD1 protein stability in THP-1 cells. Cells were treated with 25 μM CHX, with or without 1 μM STA-9090, and harvested at the predefined time points (*n* = 3). **i** Time course analysis of SAMHD1 protein in THP-1 cells. Cells were treated with 200 nM IPI-504, 200 nM PU-H71, or 20 nM STA-9090 and harvested at the indicated time points for western blot analysis. **j** MTT assay measuring THP-1 cell viability after 18-hour treatment with various doses of HSP90 inhibitors (*n* = 3). Data are presented as mean ± s.d. **k** Diagram illustrating the source organs of the cell types used. **l** Heatmap displaying SAMHD1 protein abundance in the specified cell lines after 18-hour treatment with 20 nM STA-9090, quantified using ImageJ. GAPDH functioned as a normalization control

Inhibition of HSP90 by its small-molecule inhibitors leads to the depletion of various HSP90 client proteins, including multiple oncogenic proteins.^36,42,43^ Thus, HSP90 is widely recognized as a promising molecular target for anti-cancer therapeutic strategies.^44–48^ We speculated that targeting HSP90 could be a potential way to downregulate SAMHD1 in AML cells. First, we silenced HSP90 by using multiple HSP90-specific siRNAs in THP-1 cells and discovered that HSP90 protein level affected SAMHD1 protein levels (Fig. 3a, b). Similar results were observed in Molm-13 and HEK-293T cells (Supplementary Fig. 3a, b). The clinically approved drug pimitespib^44–46^ and IPI-504 also induced the depletion of SAMHD1 in HEK293T cells and in various tumor cell types (AGS, H9, HGC-27, and HeLa) (Fig. 3c, d; Supplementary Fig. 3c, h). Consistent results were documented in the AML cell lines THP-1 (Fig. 3d, e; Supplementary Fig. 3c), Molm-13 (Supplementary Fig. 3d, e), Kasumi-1 (Supplementary Fig. 3f), and primary AML blast (Fig. 3f; Supplementary Fig. 3g). Furthermore, two other kinds of HSP90 inhibitors (PU-H71 and STA-9090) efficiently reduced SAMHD1 expression in THP-1 (Fig. 3d), Molm-13 (Supplementary Fig. 3d), and Kasumi-1 cells (Supplementary Fig. 3f) in a dose-dependent manner. Protein quantification is provided in Fig. 3e and Supplementary Fig. 3e. Except for AML cells, the efficacy of STA-9090 in downregulating SAMHD1 expression was further confirmed in other cell types from multiple solid tumors (Fig. k–l). Consistent with previous reports,^49^ HSP90 inhibition induced client-protein depletion (CDK4) with little effect on HSP90 protein levels (Supplementary Fig. 3i, j). In summary, SAMHD1 reduction following STA-9090 treatment was substantiated across 23 distinct tumor cell lines derived from nine diverse tissue types.

HSP90 inhibitor treatment did not reduce SAMHD1 mRNA levels (Fig. 3g) but showed a clear reduction in SAMHD1 protein levels (Fig. 3d) in THP-1 cells, suggesting that SAMHD1 depletion occurs at the protein level. Chase experiments using cycloheximide (an inhibitor of eukaryote protein synthesis) further indicated that the half-life of SAMHD1 protein was reduced in the presence of STA-9090 (Fig. 3h). Similar to other HSP90 client proteins,^50^ HSP90 inhibition-induced SAMHD1 depletion could also be suppressed by proteasome inhibitors (Supplementary Fig. 3k, l). Efficient SAMHD1 depletion was observed 8–16 h after treatment with HSP90 inhibitor (Fig. 3i). Under these experimental conditions, cell viability was minimally affected by the HSP90 inhibitors (Fig. 3j), suggesting that the HSP90 inhibitor-induced SAMHD1 depletion was not the result of cytotoxicity. We did observe that HSP90 inhibitor-mediated degradation of SAMHD1 impaired its previously reported role in DNA replication regulation as indicated by the enhanced expression ofγ-H2AX.^21,22,51^ Conversely, reconstitution of SAMHD1 reversed the accumulation of DNA damage marker γ-H2AX induced by the HSP90 inhibitor (Supplementary Fig. 3m, n).

**Fig. 4:**
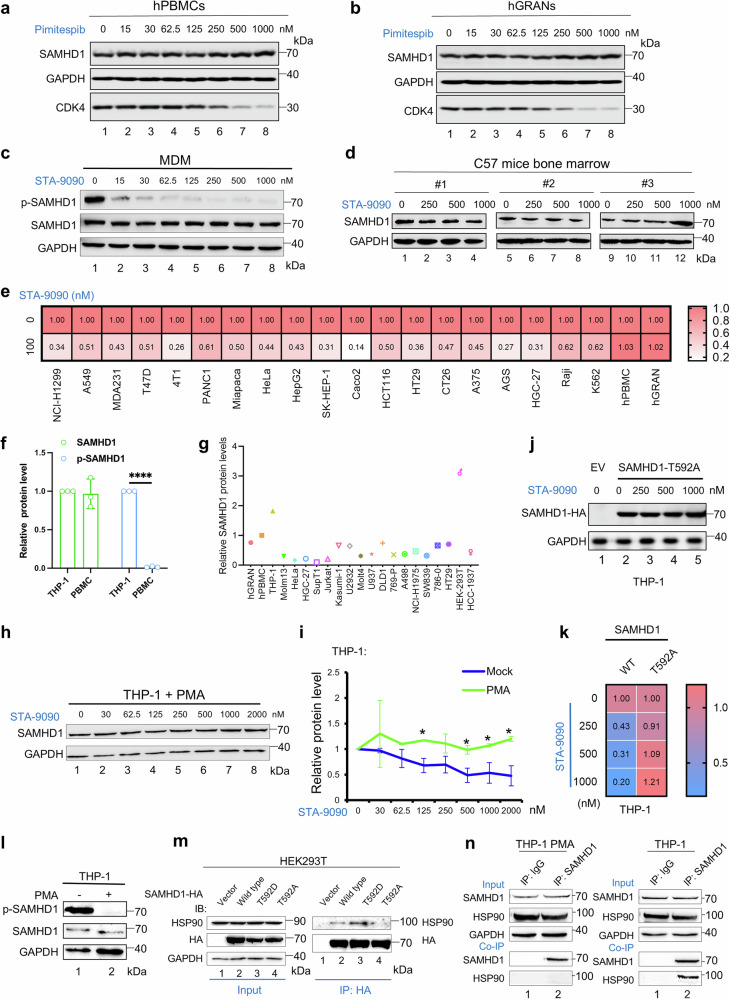
HSP90 inhibitor cannot induce depletion of SAMHD1 in healthy PBMCs, macrophages, and mice bone marrow cells. **a**, **b** Western blot analysis of SAMHD1 levels in **a** hPBMCs and **b** hGRANs from healthy donors after 18 h treatment with various doses of Pimitespib (*n* = 3 independent experiments). **c** Macrophages, differentiated from healthy donor PBMCs using CD14+ magnetic microbead isolation and 7-day culture with 10 ng/mL human GM-CSF, were treated with various doses of STA-9090 for 18 h (*n* = 3). Western blot was then employed to analyze SAMHD1 levels. **d** Western blot analysis showing SAMHD1 levels in C57 mice bone marrow were not sensitive to STA-9090 treatment for 18 h (*n* = 3). **e** Heatmap showing the relative SAMHD1 protein level across 19 different tumor cell lines and non-tumorigenic cells (hPBMCs and hGRANs) following 18 h treatment with 100 nM STA-9090. Data for each cell line were normalized to GAPDH its respective untreated control. **f** The endogenous protein levels of total SAMHD1 and p-SAMHD1 measured by Western blot and quantified by ImageJ. **g** Relative SAMHD1 levels in various cell types. Protein levels were normalized to GAPDH levels. **h** Western blot showing SAMHD1 protein expression in PMA-stimulated THP-1 differentiated macrophages after treatment with various doses of STA-9090 for 18 h (*n* = 3). **i** Line graph illustrating the relative SAMHD1 protein abundance in both undifferentiated and PMA-differentiated THP-1 cells, after 18-hour exposure to escalating concentrations of STA-9090. Data were normalized against GAPDH and their respective untreated controls. **j** Exogenous SAMHD1-T592A-HA was transduced into THP-1 cells, followed by treatment with gradually increasing doses of STA-9090 for 18 h, the level of HA-tagged SAMHD1-T592A in the cells was detected by Western blot with GAPDH as the loading control. **k** Heatmap showing the relative SAMHD1-WT or T592A protein levels in THP-1 cells following 18 h treatment of STA-9090. **l** SAMHD1 was dephosphorylated in THP-1-differentiated macrophages and phosphorylated in THP-1 cells. **m** SAMHD1 T592D, not T592A, preferentially bound to HSP90. HEK293T cells were transfected with HA-tagged wild-type SAMHD1, point mutant T592A, or T592D, then subjected to co-IP with HA beads. Western blot analysis was performed to detect precipitated proteins using an anti-HSP90 antibody (*n* = 2). **n** Co-IP analysis showing that endogenous SAMHD1 did not interact with HSP90 in THP-1 differentiated macrophages but does interact in undifferentiated THP-1 cells (using anti-SAMHD1 antibody or IgG control)

SAMHD1 is widely expressed in a variety of normal cells (Supplementary Fig. 4a), especially in immune cells, such as macrophages, dendritic cells, and lymphocytes, which mediates resistance to infection by HIV and DNA viruses.^5,6^ In contrast to the results in AML cells, HSP90 inhibitors had little effect on SAMHD1 expression in peripheral blood mononuclear cells (hPBMCs) and granulocytes (hGRAN) from healthy donors (Figs. 4a, b and S4d). Notably, the HSP90 inhibitors were functionally active in these cells, as evidenced by the significant dose-dependent decrease in CDK4, a well-known HSP90 client protein (Supplementary Fig. 3i, j). HSP90 inhibitors also had little effect on SAMHD1 expression in human macrophages, natural killer (NK) cells, and primary mesenchymal stem cells (Fig. 4c; Supplementary Fig. 4b, c). Furthermore, SAMHD1 in bone marrow cells from C57 mice was not affected by HSP90 inhibitors (Fig. 4d). In contrast, SAMHD1 depletion in the presence of HSP90 inhibitors was documented in various tumor cell types (Fig. 4e) and primary leukemia blasts derived from various AML patients (Figs. 3f and S3g), whose clinical information is provided in Supplementary Table 3. According to these results, we can conclude that SAMHD1 proteins in tumor cells, but not in multiple primary cell types, are vulnerable to HSP90 inhibition.

Interestingly, we found that SAMHD1 became resistant to the HSP90 inhibitor treatment when THP-1 cells were induced to differentiate after phorbol myristate acetate (PMA) treatment (Fig. 4h, i). PMA treatment reduced SAMHD1 phosphorylation at position 592 by more than 80% (Fig. 4l). These data suggest that phosphorylated SAMHD1 (p-SAMHD1) proteins are more sensitive to HSP90 inhibitor treatment. It has been reported that macrophages contain a small amount of p-SAMHD1 protein.^52,53^ Indeed, HSP90 inhibitor treatment resulted in efficient depletion of p-SAMHD1 protein from monocyte-derived macrophages (MDM) (Fig. 4c, anti-p-SAMHD1), whereas most of the SAMHD1 protein (unphosphorylated SAMHD1) in macrophages was resistant to treatment with HSP90 inhibitor (Figs. 4c and S4e, f, anti-SAMHD1). We compared SAMHD1 and p-SAMHD1 protein levels in PBMCs and THP-1 cells and observed comparable total SAMHD1 expression in PBMCs and THP-1 cells, whereas p-SAMHD1 expression in THP-1 cells was more than 50 times greater than that in PBMCs (Fig. 4f). Although HSP90 inhibition was able to induce depletion of wild-type SAMHD1 in THP-1 cells (Fig. 3d), we discovered that HSP90 inhibition had little effect on the expression of SAMHD1 mutant T592A (Fig. 4i–k), which is unable to be phosphorylated at this position.^54^ We found that in THP-1 cells, both STA-9090 and pimitespib effectively downregulated SAMHD1 and p-SAMHD1 levels. The downregulation extent of total SAMHD1 and p-SAMHD1 was similar at each drug dose (Supplementary Fig. 4g, h), suggesting that most of the SAMHD1 protein in THP-1 cells existed as the phosphorylated form. The T592 position phosphorylation status has been shown to alter SAMHD1 intracellular distribution,^55^ thus altered interaction of phosphorylated and un-phosphorylated SAMHD1 proteins with HSP90 may be partially explained by their cellular location. Furthermore, SAMHD1 containing a phosphomimetic aspartic acid residue (T592D) interacted to a much greater extent with HSP90 than the non-phosphorylatable SAMHD1 mutant T592A (Fig. 4m). Endogenous SAMHD1 interacted with HSP90 in mock THP-1 rather than in PMA-induced THP-1 cells (Fig. 4n). Collectively, these data indicate that p-SAMHD1 proteins are the main target of HSP90 inhibitors.

### HSP90 inhibitor and ara-C exert synergistic effect against AML cells in vitro

**Fig. 5:**
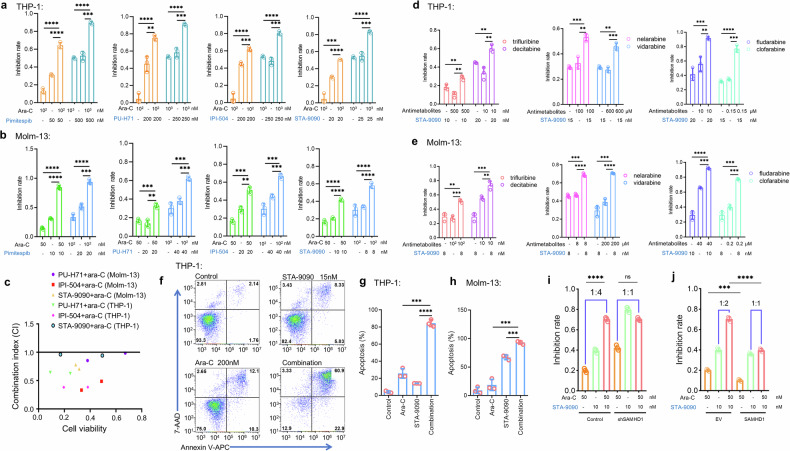
HSP90 inhibitors and ara-C exert a synergistic effect in killing AML cells. **a**, **b** Bar graphs illustrating the inhibition efficacy of ara-C and multiple HSP90 inhibitors (used as single agents or in combination) on **a** THP-1 cells and **b** Molm-13 cells following 72-hour exposure. Cell viability was assessed via MTT assay (*n* = 3). **c** Combination Index (CI) values for THP-1 and Molm-13 cells treated with ara-C plus IPI-504, PU-H71, or STA-9090. Drug interaction was evaluated using CompuSyn software via the Chou-Talalay method: CI < 1 denotes synergism, CI = 1 indicates an additive effect, and CI > 1 signifies antagonism. **d**, **e** Synergistic enhancement of cytotoxicity of other antimetabolites (fludarabine, and clofarabine), when combined with STA-9090, demonstrated in **d** Molm-13 (*n* = 3) and **e** THP-1 (*n* = 3). **f**, **g** Apoptosis analysis in THP-1 cells treated with STA-9090 and ara-C alone or in combination for 72 h. Apoptosis was assessed by flow cytometry after staining with 7-AAD and annexin V (*n* = 3). **g** Quantification of apoptosis rates from the experiment in (**f**). **h** Apoptosis rates of Molm-13 cells exposed to STA-9090 and ara-C as single agents or in combination for 72 h (*n* = 3). **i** Cytotoxicity analysis of shSAMHD1-transduced and control Molm-13 cells treated with ara-C and HSP90 inhibitors either alone or combined for 72 h (*n* = 3). **j** Cytotoxicity assay of Molm-13 transduced with exogenous SAMHD1 or empty control treated with ara-C and HSP90 inhibitors alone or in combination for 72 h. Cell viability was measured by MTT (*n* = 3). Data are shown as mean ± s.d. *p < 0.05; **p < 0.01; ***p < 0.001; ****p < 0.0001

It has been reported that SAMHD1 is an ara-C-resistant factor in AML cells.^32,33^ We also found that silencing SAMHD1 with shRNA in THP-1 and Molm-13 cells enhanced the ara-C-induced cytotoxicity (Fig. 1c–f). Considering that HSP90 inhibitors trigger SAMHD1 depletion in AML cells, we evaluated the synergistic effect of the combined ara-C and HSP90 inhibitor treatment in THP-1 and Molm-13 cells. Dose–response curves of THP-1 and Molm-13 cells to different HSP90 inhibitors are provided in Supplementary Fig. 5a and b. Compared with any single-drug treatment, the combination of HSP90 inhibitors (pimitespib, IPI-504, PU-H71, or STA-9090) with ara-C significantly improved the cytotoxicity of ara-C against THP-1 and Molm-13 cells as measured by the MTT assay (Fig. 5a, b). Combination index (CI) analysis also showed a synergistic effect of these four HSP90 inhibitors in combination with ara-C (Fig. 5c). However, the synergistic effect was attenuated when SAMHD1 was silenced (Fig. 5i). Overexpression of SAMHD1 not only inhibited the cell-killing effect of ara-C, but it also abrogated the synergistic effect of ara-C and HSP90 inhibitors (Fig. 5j). This demonstrates that the synergistic effect depends on HSP90-mediated degradation of SAMHD1, not the other HSP90 client proteins.

Given its greater effect on SAMHD1 downregulation compared with the other two HSP90 inhibitors (Fig. 3), we chose STA-9090 for further investigation. Light microscopic images were consistent with the MTT results, revealing the fewest cells in the combination group (Supplementary Fig. 5c–e). Furthermore, the combination treatment with these two drugs increased the percentage of apoptosis in both THP-1 (Fig. 5f, g) and Molm-13 (Supplementary Fig. 5f, g) cells, compared with single-drug treatment. The decrease in SAMHD1 expression caused by STA-9090 was also confirmed in the presence of ara-C in THP-1 and Molm-13 cells (Supplementary Fig. 5g). The concentrations of HSP90 inhibitors used in our experiments were far below the plasma concentration of treated patients in clinical trials.^56–58^ We found that SAMHD1 overexpression in molm-13 cells diminished the synergistic effect of Ara-C and HSP90 inhibitor-induced apoptosis (Supplementary Fig. 5h–j). In addition to ara-C, the HSP90 inhibitor STA-9090 enhanced the antitumor effect of other antimetabolites, including vidarabine (used for treatment of acute keratoconjunctivitis and recurrent epithelial keratitis), clofarabine (used for all tumors), decitabine (used for myelodysplastic syndrome and AML), nelarabine (used for T-cell acute lymphoblastic leukemia), fludarabine (used for leukemia and lymphoma), and trifluridine (used for epithelial keratitis and colorectal cancer) in THP-1 and Molm-13 cells (Fig. 5d, e). These results suggest that HSP90 inhibitors have the potential to enhance the antitumor effect of other antimetabolites, especially in tumors highly expressing SAMHD1.

### STA-9090 enhances the efficacy of ara-C in both heterotopic and orthotopic AML mouse models

**Fig. 6:**
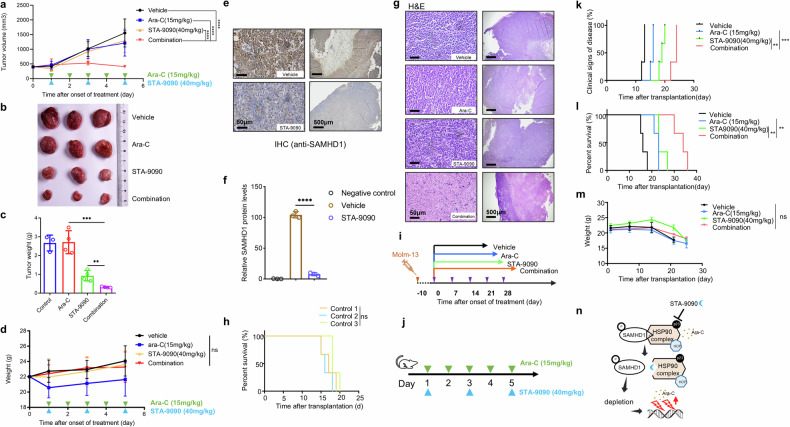
Combined treatment of STA-9090 and ara-C leads to AML tumor regression in heterotopic and orthotopic AML mouse models. NOD-Prkdcscid IL2rgtm1/Bcgen (B-NDG) mice were subcutaneously injected with 6 × 10^6^ Molm-13 cells. Drug treatment begun once the tumor volume reached 400 mm^3^. **a** Tumor growth curve during the treatment, showing STA-9090 significantly strengthened the repression of tumor growth induced by ara-C in the combination group. The tumor volume was measured by caliper every 2 days. Data represent mean ± s.d. (*n* = 3 mice per treatment group). **b** Representative macroscopic images of tumors dissected from different groups of mice at the experiment endpoint. **c** Mean tumor weight of different treatment groups at the experiment endpoint. **d** Average body weights of mice across various groups over the treatment period, confirming that treatment with either drug did not produce additional systemic toxicity (ns, not significant). **e**, **f** Representative microscopic images of anti-SAMHD1-stained IHC excised tumor sections of vehicle and STA-9090 treated groups. Relative SAMHD1 protein levels were measured by imageJ. **g** Representative H&E-stained sections showing that the drug combination caused massive karyolysis and broad tumor cell necrosis. Images were acquired by an mshot TV0.63XC-M0. **h** B-NDG mice were intravenously injected with 1 × 10^6^ Molm-13 cells to establish the systematic AML model. Three independent experiments indicated the stabilization of our orthotopic model (*n* = 3 mice per group). Ns, not significant. **i** Treatment regimen details for orthotopic mouse model. Treatment began after 10 days of engraftment and included several cycles. **j** Treatment schedule for heterotopic mouse model. Green arrowheads indicate daily 15 mg/kg ara-C treatment, and blue arrowheads indicate 40 mg/kg STA-9090 twice a week. Treatment with vehicle, 15 mg/kg ara-C by daily subcutaneous injection, or 40 mg/kg STA-9090 via tail-vein injection (on days 3 and 6, indicated by blue arrowheads) began on day 0. **k** Occurrence rate of clinical manifestations of disease (hind-limb paralysis) over the treatment duration in the orthotopic mouse model. **l** Kaplan–Meier plots of the mice with AML (*n* = 3 mice/group). Survival differences were assessed by Mantel–Cox log-rank test. *p < 0.05; **p < 0.01; ***p < 0.001; ****p < 0.0001. **m** Body weight of the AML orthotopic mouse model throughout the treatment period. **n** Schematic summarizing how HSP90 inhibitors induce SAMHD1 depletion in AML, resulting in increased anti-leukemic activity when combined of ara-C

The synergistic effect of ara-C and HSP90 inhibitor in vivo was further evaluated in mouse models. We chose STA-9090 to combine with ara-C because it was more effective in vitro than IPI-504 or PU-H71, and it has been reported to exhibit high potency in preclinical tumor models, with favorable activity and tolerability compared with IPI-504 and PU-H71.^59–61^ First, we evaluated the effect of drug treatments on tumor growth in a xenotransplantation mouse model by subcutaneously injecting Molm-13 cells into immunodeficient NOD-Prkdcscid IL2rgtm1/Bcgen (B-NDG) mice. To test the effect of the combination therapy, we commenced treatment after tumor volume had reached a threshold of 400 mm^3^. The mice (randomly grouped) were treated intratumorally with vehicle (untreated control group) or with 15 mg/kg ara-C alone, 40 mg/kg STA-9090 intravenously alone, or the ara-C/STA-9090 combination. The experiment was terminated when the mean tumor length reached 2 cm in the untreated mice. The combination treatment with ara-C and STA-9090 completely halted the tumor growth compared with the untreated group or the groups treated with ara-C or STA-9090 alone (Fig. 6a). The average tumor weight (volume) at the end of the experiment was significantly lower in the group that had received the combination treatment than in the control group or the groups treated with ara-C or STA-9090 alone (Fig. 6b, c). All four of the groups of mice had comparable total body weights throughout the experiment (Fig. 6d), indicating that ara-C alone, STA-9090 alone, or their combination had no detectable toxicity in vivo. In addition, immunohistochemical analysis (Fig. 6e, f) demonstrated that STA-9090 treatment reduced the expression of SAMHD1 in the Molm-13 xenograft tumors, consistent with our in vitro data (Supplementary Fig. 3d). Moreover, hematoxylin and eosin (H&E) staining of the residual tumor mass in the mice treated with both STA-9090 and ara-C revealed massive necrosis, broad nuclear karyorrhexis, and karyolysis, whereas the mice receiving STA-9090 alone displayed only some nuclear pyknosis and those treated with ara-C alone showed no histological changes (Fig. 6g).

Next, we established the systemic spread of the leukemia by tail-vein injection of 1 × 10^6^ Molm-13 cells into NOD-Prkdcscid IL2rgtm1/Bcgen (B-NDG) mice (Fig. 6h–m). We found that the onset of hind-limb paralysis was a sign of disease progression that preceded death; thus, it was used to assess the clinical stage of the disease. After 10 days of engraftment, the mice were randomly divided into four groups, which were treated as shown in Fig. 6i (experimental groups: a. vehicle group, b. 15 mg/kg ara-C daily for 7 days via subcutaneous injection, as indicated by blue arrowheads in Fig. 6j; c. 40 mg/kg STA-9090 twice a week via intravenous injection, as indicated by green arrowheads in Fig. 6j; d. both ara-C and STA-9090 treatment as described above) until the mice succumbed or reached an experimental end point. The onset of clinical signs of disease appeared much earlier in the groups that received vehicle or single drug (ara-C or STA-9090) than in the group receiving the ara-C/STA-9090 combination (Fig. 6k). Furthermore, the combination therapy with ara-C and STA-9090 significantly prolonged their survival compared with other groups (Fig. 6l), without causing extra weight loss (Fig. 6m). In addition, even if we postponed treating the combination group for one week after we began the single-drug treatments, we still observed a later onset of disease progression and a longer survival in the combination-treated group (Supplementary Fig. 6a, b).

### STA-9090 triggers innate immune responses in tumor cells and enhances antitumor effect of immune cells

**Fig. 7:**
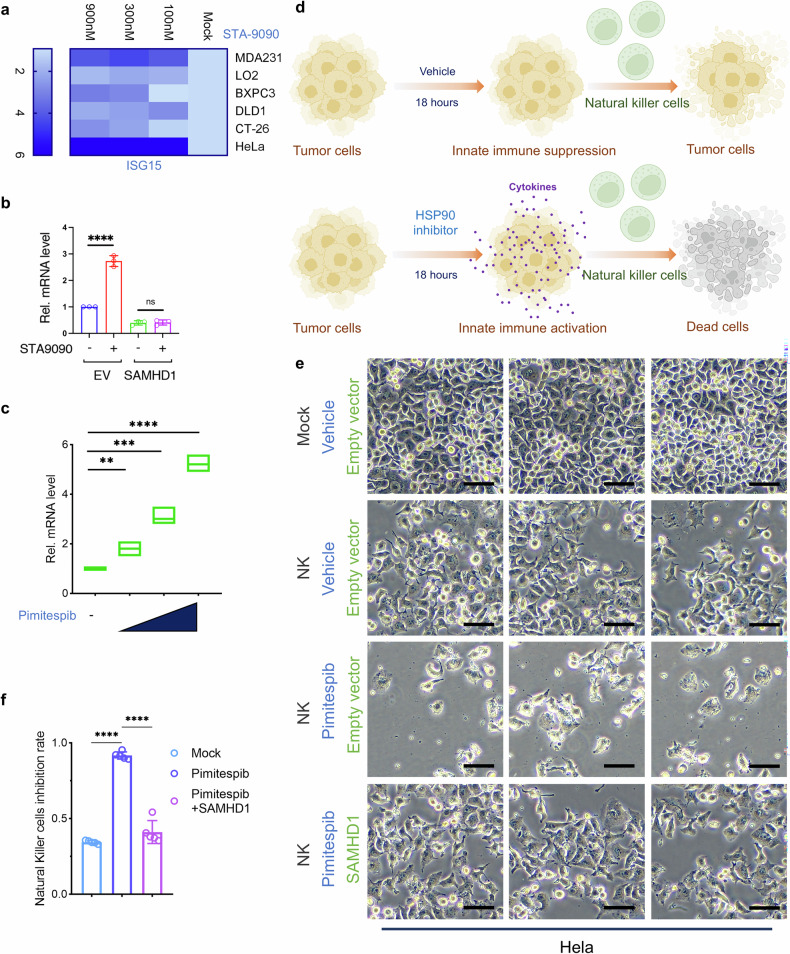
STA-9090 triggers innate immune responses in tumor cells and enhances antitumor effect of immune cells. **a** Dose-dependent induction of ISG15 expression by STA-9090. Indicated cell lines were treated with escalating doses of STA-9090 (15 nM, 125 nM, and 1000 nM) for 18 h. ISG15 protein expression was assessed by western blot, with GAPDH serving as a loading control. Quantification of ISG15 protein levels, normalized to GAPDH, was performed using ImageJ. **b** RT-qPCR assay to detect IFNB mRNA expression in HeLa cells transduced with SAMHD1 and empty vector following 18-hour exposure to STA-9090 (*n* = 3). Data are expressed as mean ± s.d. **c** RT-qPCR analysis of IFNB mRNA levels in HeLa cells after 18-hour treatment with increasing Pimitespib concentrations (*n* = 3). Data represent mean ± s.d. **d** Schematic showing the mechanism of HSP90 inhibitors enhancing NK cell-mediated tumor killing. **e** Typical phase-contrast microscopic images illustrating morphological alterations in HeLa cells exposed to Pimitespib as monotherapy or in combination with NK cells (at an E:T ratio of 10:1) following 24-hour incubation. Scale bar, 100 μm. **f** Quantitative assessment of NK-cell cytotoxic activity. Target cell viability was determined by living cell staining and counting following 24-hour co-culture (*n* = 3). Data was normalized to untreated control and shown as mean ± s.d. *p < 0.05; **p < 0.01; ***p < 0.001

Recently, p-SAMHD1 proteins have been linked to innate immune suppression given that they been demonstrated to remove intracellular DNA and prevent cGAS-STING activation; furthermore, SAMHD reduction triggers innate immune activation.^22,24^ Triggering innate immune activation in tumors is beneficial for the antitumor host immune responses.^62,63^ We found that STA-9090 treatment led to obvious depletion of SAMHD1 and the activation of interferon-stimulated gene 15 (Fig. 7a). Further, innate immune activation, including interferon β, IFIT1, and inflammatory factor IL-1β, was observed in HeLa tumor cells (Figs. 7c and S7a–c). Overexpression of wild-type SAMHD1 suppressed HSP90 inhibitor-induced innate immune activation in HeLa cells, establishing HSP90 inhibitor-induced SAMHD1 degradation as the principal driver of this immunostimulatory effect (Fig. 7b). In addition, NK cells and spleen-derived immune cells were able to induce tumor-cell death, and this effect was enhanced in the presence of HSP90 inhibitors (Figs. 7d, e and S7d–h). Exogenous supplementation of SAMHD1 expression abrogated the promotion of NK-cell cytotoxicity by HSP90 inhibitors (Fig. 7e, f).

## Discussion

SAMHD1 expression is commonly elevated in various types of cancer, and its increased levels are associated with poor patient survival (Fig. 1; Supplementary Fig. 1). Increased SAMHD1 levels also contribute to the resistance against chemotherapeutic treatments, particularly those involving nucleoside analogs, such as cytarabine (ara-C) in the context of acute myeloid leukemia (AML).^64,65^ SAMHD1’s role in regulating intracellular dNTP levels is central to its ability to protect tumor cells from the cytotoxic effects of chemotherapy. In contrast, SAMHD1 expression in normal cell types, particularly in lymphoid cells, plays a crucial role in protecting against viral infections.^5^ This presents a significant challenge for selectively depleting tumor-associated SAMHD1 without affecting its normal cellular functions. In this study, we introduced a novel approach to selectively and efficiently deplete SAMHD1 in various tumor cells while preserving its expression in normal cells. We demonstrated that SAMHD1 protein levels in tumor cells are regulated by HSP90 complexes. Treatment with various HSP90 inhibitors, including the clinically approved drug Pimitespib, resulted in efficient SAMHD1 depletion in a variety of tumor cell types (Fig. 3; Supplementary Fig. 3). Importantly, SAMHD1 levels in key normal primary cells, such as human macrophages, lymphoid cells, and mouse bone marrow cells, were either unaffected or only minimally affected by HSP90 inhibition (Fig. 4; Supplementary Fig. 4). This finding suggests that targeting SAMHD1 via HSP90 inhibition is a promising strategy that may minimize the risks associated with nonselective SAMHD1 inhibition.

SAMHD1 is known to contribute to resistance against antimetabolites, such as the first-line chemotherapy drug cytarabine (ara-C) in AML patients. Previous studies have shown that targeting SAMHD1 can enhances the efficacy of ara-C treatment in AML by lowering dNTP levels, which in turn sensitizes AML cells to chemotherapy.^32,65,66^ Our study demonstrated that HSP90 inhibitors synergize with ara-C, not only in vitro but also in orthotopic and heterotopic AML mouse models in vivo. These findings align with previous studies showing that reducing SAMHD1 expression improves ara-C response.^67,68^ Our data support a novel personalized approach to enhance the anti-cancer effects of ara-C by combining it with HSP90 inhibitors. Beyond ara-C, we found that the anti-tumor efficacy of several clinically used drugs, including trifluridine, decitabine, fludarabine, and clofarabine, was significantly enhanced when combined with HSP90 inhibitors (Fig. 5d, e). These drugs are commonly used to treat various cancers, including colon cancer, lung cancer, chronic myelomonocytic leukemia (CMML), multiple myeloma (MM), chronic lymphocytic leukemia (CLL), acute lymphoblastic leukemia (ALL), and non-Hodgkin lymphoma. Therefore, HSP90 inhibitors may have clinical benefits for the treatment of other tumors, especially those in which SAMHD1 is highly expressed and associated with poor patients’ survival (Supplementary Fig. 8).

In rapidly proliferating tumor cells, SAMHD1 is phosphorylated at threonine 592, whereas in quiescent lymphoid cells and macrophages, SAMHD1 is predominantly in a dephosphorylated state.^54^ Previous studies have shown that the phosphorylation status of SAMHD1 threonine 592 does not affect its resistance to ara-C.^32^ Therefore, targeting tumor-specific SAMHD1 should primarily focus on phosphorylated-SAMHD1 (p-SAMHD1). Our data suggest that the phosphorylation status of SAMHD1 may be a key factor in selective sensitivity of tumor-associated SAMHD1 compared to SAMHD1 proteins in normal cells when treated with HSP90 inhibitors. In cycling THP-1 cells, SAMHD1 was predominantly phosphorylated and could be downregulated by HSP90 inhibitors. However, in quiescent THP-1, where SAMHD1 became largely dephosphorylated, it was no longer responsive to HSP90 inhibitors (Fig. 4h, i). Additionally, we observed that the small amount of p-SAMHD1 protein in primary macrophages was more sensitive to HSP90 inhibitors than the largely unphosphorylated SAMHD1 in these cells (Fig. 4c). These results suggest that the phosphorylation status of SAMHD1 may play an important role in its selective depletion in tumor cells, as phosphorylated SAMHD1 appears to be more susceptible to HSP90 inhibition compared to its unphosphorylated counterpart in normal cells. The p-SAMHD1 complexed with HSP90 may play a crucial role in tumor cells. It has been reported that the p-SAMHD1 recruit meiotic recombination 11 (MRE11) to remove intracellular DNA fragments, thereby preventing activation of the cGAS-STING pathway.^22^ Activation of the cGAS-STING pathway leads to the production of type-I interferons and other inflammatory cytokines, which can enhance anti-tumor immune responses. In our study, we found that HSP90 inhibitors induced SAMHD1 depletion, leading to the activation of innate immune responses and triggering apoptosis in tumor cells. The depletion of SAMHD1 by HSP90 inhibitor, alongside the triggering of the cGAS-STING pathway, may not only contribute to tumor cell apoptosis but also enhance the anti-tumor effect of ara-C in AML cells. These results point to the potential of combining SAMHD1-targeting therapies with immune modulation to improve treatment outcomes in cancer therapy.

It is widely recognized that SAMHD1 phosphorylation at position 592 impairs its anti-HIV function.^69,70^ However, the exact mechanism behind this impairment remains unclear. One possibility is that SAMHD1 may interact with HIV components, such as the viral protein CAp24, to mediate viral restriction.^10^ We have shown that phosphorylated SAMHD1 molecules are associated with HSP90 complexes, which may prevent SAMHD1 from interacting with HIV component and inhibiting the virus. In contrast, unphosphorylated SAMHD1 is not bound to HSP90 complexes, allowing it to freely interact with HIV components and perform viral inhibition. Future studies will be needed to explore this intriguing hypothesis, as understanding the interaction between SAMHD1 and HIV components could provide valuable insights into viral inhibition and the development of new antiviral therapies.

Our strategy for targeting SAMHD1 differs from general SAMHD1 inhibition in that it selectively targets tumor cells. A major concern with nonselective SAMHD1 inhibition is that SAMHD1 is widely expressed in various normal cells, including hematopoietic stem cells. Combining a SAMHD1 inhibitor with ara-C could lead to excessive toxicity, particularly myelosuppression, which is a common side effect of chemotherapy. Additionally, unphosphorylated SAMHD1 plays a crucial role in maintaining a low intracellular dNTP levels in quiescent cells, including macrophages and quiescent CD4 + T cells, where it mediates viral restriction.^1,7,52,53^ Altered dNTP levels could result in DNA damage and aberrant replication, potentially leading to tumorigenesis.^57^ SAMHD1 also has key roles in innate immune regulation and DNA damage responses.^22^ Therefore, it is important to preserve SAMHD1 function in normal resting cells when selectively targeting SAMHD1 in tumor cells. The approach of selectively depleting SAMHD1 in tumor cells, as described in this study, holds promising potential for future therapeutic applications.

## Materials and methods

### Study approval

All experimental animal procedures were performed in compliance with the Chinese. The study was conducted in accordance with the Laboratory Animal Guidelines for Ethical Review of Animal Welfare and was approved by the Zhejiang Chinese Medical University Experimental Animal Management and Ethics Committee (ZSLL-2018-19).

Healthy human PBMCs were obtained from recruited donors with the approval of the regional ethics committee of the Second Affiliated Hospital of Zhejiang University, School of Medicine (G2018-200).

### Cell culture and patient samples

Human hematological malignancy cell lines THP-1, Kasumi-1, Molm-13, and H9; human solid tumor cell lines AGS, HGC-27, HeLa, HEK293T, A549, HCT-29, and HT-116 were purchased from the American Type Culture Collection (ATCC) and cultured in accordance with the ATCC’s recommendations. THP-1, Kasumi-1, H9, AGS, HGC-27, HCT-116, and HT-29 cells were grown in RMPI-1640 medium (Gibco) containing 10% fetal bovine serum (FBS; BI), 100 IU/ml streptomycin, and 100 IU/ml penicillin (TBD Science). 293T, A549, and HeLa cells were cultured in DMEM medium (Gibco) with 10% FBS, 100 IU/ml streptomycin, and 100 IU/ml penicillin. Molm-13 and primary leukemia blasts were cultured in IMDM medium (GSEE-TECH) with 10% FBS, 100 IU/ml streptomycin, and 100 IU/ml penicillin. All cells were kept within a humidified incubator at 37 degrees Celsius with 5% CO₂. PMA (200 nM) was used to induce differentiation of THP-1 cells for 16–24 h before initiating drug treatment.

Primary AML samples were obtained from the Second Affiliated Hospital of Zhejiang University, School of Medicine and were approved by the local ethics committee (G2018-200).

#### Monocyte purification and differentiation

Healthy peripheral blood mononuclear cells were isolated from enrolled donors, and all experimental protocols were approved by the regional ethics committee of the Second Affiliated Hospital of Zhejiang University, School of Medicine (G2018-200). The cells were isolated using a Ficoll-Hypaque density gradient (TBD Sciences) and cultured in RMPI-1640 medium under the same conditions as the AML cell lines. Monocytes were separated and enriched by CD14+ magnetic microbeads (MCS), then differentiated into macrophages by culturing for 7 days in RMPI 1640 supplemented with 10% FBS, 100 IU/ml penicillin, 100 IU/ml streptomycin, and 10 ng/ml human granulocyte macrophage colony stimulating factor (GM-CSF) (R&D Systems) after washing to remove the non-adherent cells.

### Plasmids, reagents, and antibodies

SAMHD1 and HSP90 wild-type (WT), point mutants, and truncation constructs were cloned into VR1012 as previously described.^2,56^ IPI-504 (Retaspimycin), STA-9090 (Ganetespib), PU-H71, nelarabine, clofarabine, vidarabine, decitabine, trifluridine, fludarabine, and cycloheximide were commercially obtained from Medchemexpress (MCE) and dissolved in dimethyl sulfoxide (DMSO); cytarabine (Ara-C) was obtained from Selleckchem Ltd. and dissolved in phosphate buffer saline (PBS). The following antibodies were used in this study: anti-SAMHD1 (Origene), anti-phospho-SAMHD1 (Thr592) (Prosci), Anti-phospho-Histone H2A.X (Ser139) (20E3) Rabbit mAb #9718 (CST). Anti-HA (Invitrogen), anti-HSP90 (ET1605-56), anti-GAPDH, and goat anti-rabbit/mouse horseradish peroxidase (HRP)-conjugated and FITC/rhodamine-conjugated secondary antibodies (Huabio).

### Drug screening

To identify small-molecule drugs capable of effectively downregulating the SAMHD1 protein expression, we conducted functional screening of 122 small-molecule drugs (MCE; Supplementary Table 1) covering multiple cellular signaling pathways. The effects of drugs on SAMHD1 expression were assessed through western blot analysis and their levels were quantified via ImageJ.

### Cytotoxicity assays

AML cells were seeded in triplicate into 96-well plates at a final density of 8000 cells/well and treated with various drug concentrations. Following a 72 h incubation at 37 °C, cellular viability was measured with a 3-(4,5-dimethylthiazol-2-yl)-2,5-diphenyltetrazolium bromide (MTT) kit (Sangon Biotech) as per the supplier’s instructions. Absorbance was measured at a wavelength of 570 nm (with a 630 nm reference wavelength) using a Molecular Devices SpectraMax Absorbance Reader. IC_50_ values were calculated using Prism 6 (GraphPad software).

Drug synergy was evaluated using the Chou-Talalay method and CompuSyn software.^71^ We calculated the CI values based on data from non-constant ratio drug combination experiments. CI < 1 denotes synergism, CI = 1 signifies an additive effect, and CI > 1 indicates antagonism.

### Extraction of proteins and Western blot assay

Cell pellets were lysed in Radioimmunoprecipitation assay (RIPA) buffer (Beyotime Biotechnology) on ice with the addition of 0.1% (100 mM) of phenylmethylsulfonyl fluoride (PMSF) protease inhibitor (FDbio Sciences). For tumor tissue samples, approximately 100 mm^3^ tumor mass was dissected out and homogenized in RIPA buffer on ice.

The concentration of total protein was assayed by means of the Bradford assay (Bio-Rad). Equal amount of protein extracts were loaded onto denaturing 10% SDS-polyacrylamide electrophoresis (PAGE) gels and separated electrophoretically, before being transferred to nitrocellulose membranes (GE Whatman). Following this, the membranes were blocked with 5% skim milk (BD Biosciences), incubated with primary antibodies at 4 °C overnight, then with matching secondary antibodies at room temperature for 1 h. Protein bands were detected using enhanced chemiluminescence (ECL) reagents (Yeasen) on a Tanon 5200 imaging system; their intensities were quantified with ImageJ software (v1.53).

### Co-immunoprecipitation

Cell populations were lysed using low-strength RIPA buffer (EMD Millipore) with the addition of protease inhibitor cocktail (Roche) for 2 h at 4 °C, then centrifuged at 13,000 × *g* for 20 min to clarify the proteins.

Protein A/G magnetic beads (Bimake) were incubated with either a specific antibody or an equivalent concentration of control IgG for 20 min at room temperature to create antibody-coated beads. These beads were then added to the cell lysate and incubated at 4 °C overnight. The magnetic beads were undergoing three washes with wash buffer (PBS with 0.2% Triton X-100). Bound proteins were then eluted by boiling the beads with 1x loading buffer (FDBio Sciences), and the immunoprecipitants were detected by immunoblotting.

HEK293T cells were subjected to transfection with the HA-tagged plasmid via Lipofectamine 2000 (Invitrogen) and were harvested 48 h later. Total proteins were extracted using the method described above and incubated together with HA-conjugated agarose beads (Roche) overnight. The agarose beads were then washed 4–5 times with wash buffer and eluted using glycine buffer (0.2 M glycine) for mass spectroscopy (MS) analysis or boiled with loading buffer for Western blot assay.

### Mass spectrometry

Samples underwent sonication-mediated lysis (three cycles on ice) using a high-intensity ultrasonic processor (Scientz) in lysis buffer (8 M urea, 1% protease inhibitor cocktail). Following this, insoluble debris was sedimented by means of centrifugation at 12,000 × *g* for 10 min at 4 °C. After collecting the supernatant, protein concentration was assayed via a BCA kit (Bicinchoninic Acid; Beyotime) following the manufacturer’s recommended procedures.

For subsequent digestion, protein samples were subjected to reduction with 5 mM dithiothreitol at 56 °C for 30 min, and then to alkylation using 11 mM iodoacetamide at room temperature for 15 min in the dark. The samples were then diluted by adding 100 mM triethylammonium bicarbonate (TEAB) to a final urea concentration of less than 2 M. Trypsin was added at a 1:50 trypsin-to-protein mass ratio for overnight primary digestion, and at a 1:100 ratio for a 4-hour secondary digestion. The resulting peptides were then desalted with a C18 SPE column.

For liquid chromatography (LC) separation, tryptic peptides were dissolved in solvent A (0.1% formic acid in water) and directly loaded onto an in-house packed reversed-phase analytical column (15-cm length, 100 μm i.d.). The separation process was implemented on a Vanquish Neo UPLC system (ThermoFisher Scientific) at a constant flow rate of 400 nL/min. Mobile phases included solvent A and solvent B (0.1% formic acid, 80% acetonitrile in water), with the gradient program as follows: 4% B (0–0.5 min); 4%–8% B (0.5–0.6 min); 8%–22.5% B (0.6–13.6 min); 22.5%–35% B (13.6–20.5 min); 35%–55% B (20.5–20.9 min); 55%–99% B (20.9–21.4 min); 99% B (21.4–22.6 min).

For mass spectrometric analysis, separated peptides were analyzed on an Orbitrap Astral MS (ThermoFisher Scientific) equipped with a nano-electrospray ion source (1900 V operating voltage). The Orbitrap detector was used for precursor analysis, while the Astral detector handled fragment assessment. Full MS scans were set to a resolution of 240,000 over a 380–980 *m*/*z* range. MS/MS scans were acquired at 80,000 resolution, with a fixed initial mass of 150.0 *m*/*z*. HCD fragmentation employed a normalized collision energy (NCE) of 25%, and the automatic gain control (AGC) target was adjusted to 500%, with a 3 ms maximum injection time.

DIA-NN search engine (v1.8) was utilized for processing the generated DIA data. Tandem mass spectra were searched against the *Homo sapiens* database (FASTA file: Homo_sapiens_9606_SP_20241202.fasta, 20,422 entries) concatenated with reverse decoy database. Trypsin/P served as the specified cleavage enzyme (allowing up to one missed cleavage) for database queries. Fixed modifications included N-terminal methionine excision and Cys carbamidomethylation, while the FDR was adjusted to <1%. Protein-protein interactions of differentially expressed protein accessions were analyzed using the STRING database, and Cytoscape was employed to visualize the interaction network.

### Immunofluorescent staining

AML cells (THP-1 and Molm-13) were cultured on cover glasses, fixed with 4% paraformaldehyde, and then permeabilized with 0.1% Triton X-100 for 20 min at room temperature. After blocking with 3% bovine serum albumin (BSA) for 30 min, the cells were incubated with primary antibodies (anti-SAMHD1 or anti-HSP90), followed by their corresponding FITC- or rhodamine-conjugated secondary antibody. Finally, coverslips were mounted using fluoromount-G containing DAPI (SouthernBiotech) for nuclear staining and visualization with a fluorescent microscope (ZEISS Axio). Images were acquired with a ZEISS Confocal Laser Scanning Microscope 710 (LSM710).

### Tissue staining

For histological sample preparation, tissues were fixed in 4% paraformaldehyde over 24 h, prior to undergoing standard dehydration and paraffin embedding. The embedded blocks were cut into 4-μm sections and mounted on glass slides. Subsequent to deparaffinization and rehydration, the tissue slices were stained with H&E (hematoxylin and eosin) for histological assessment, following standard procedures. For immunohistochemistry (IHC), sections first underwent endogenous peroxidase-blocking and antigen retrieval. The tissue slices were then incubated with anti-SAMHD1 antibody for 1 h at room temperature. Immunodetection was performed using a diaminobenzidine (DAB) substrate per the manufacturer’s recommendations. Images were acquired by a NIKON DS-U3 vision system equipped with an mshot TV0.63XC-M0.

### RT-qPCR

For gene expression quantification, total RNA was extracted from cells using the RNeasy mini Kit (Qiagen), and then reverse-transcribed into cDNA via the PrimeScript RT Master Mix (Takara). Next, quantitative real-time PCR was performed utilizing SYBR Green Master Mix (Takara) on a 7500 Fast Real-Time PCR system.

Primers used are as follows: *SAMHD1*-F: 5’-TCCATCCCGACTACAAGACA-3’, *SAMHD1*-R: 5’-TCTCGGATGTTCTTCAGCAG-3’, *GAPDH*-F: 5’-TCGACAGTCAGCCGCATCT-3’, *GAPDH*-R: 5’-CTTGACGGTGCCATGGAATT-3’, *IFIT1*-F: 5’-TTGATGACGATGAAATGCCTGA-3’, *IFIT1*-R: 5’-CAGGTCACCAGACTCCTCAC-3’, *IFNB1*-F: 5’-CGCCGCATTGACCATCTA-3’, *IFNB1-*R: 5’-GACATTAGCCAGGAGGTTCT-3’, *IL1B*-F: 5’-ATGATGGCTTATTACAGTGGCAA-3’, *IL1B*-R: 5’-GTCGGAGATTCGTAGCTGGA-3’.

### RNA silencing

To knock down HSP90/SAMHD1 expression, pLKO.1 (Addgene, #8453)^46^ based lentivirus transduction system was used. Stable shHSP90 and shSAMHD1 HEK293T, and shHSP90 and shSAMHD1 Molm-13 cell lines were subsequently established under puromycin (1 μg/ml) selection for 2 weeks. The sequences of shRNA oligos are provided in the Supplementary Materials.

### Flow cytometry

For apoptosis evaluation, AML cells were harvested, followed by staining with the Annexin V-APC/7-AAD apoptosis detection kit (MultiSciences) following the supplier’s recommended protocols. Data acquisition was performed on a Beckman Coulter flow cytometer, and the results were analyzed with FlowJo (Version X.07).

### Orthotopic AML animal model

AML cells (Molm-13, 1 × 10^6^ cells/mouse) were transplanted into 7-week-old female NOD-Prkdc^scid^ IL2rg^tm1^/Bcgen (B-NDG) mice (Biocytogen, Beijing, China)^47^ by tail vein injection. Following a 10-day engraftment period, mice were randomly assigned to distinct treatment groups in preparation for subsequent therapeutic interventions. Mice were treated daily with 15 mg/kg cytarabine via subcutaneous injection or 40 mg/kg STA-9090 formulated in 10/18 DRD (10% DMSO, 3.6% D-glucose and 18% Cremophor RH 40 in water) via intravenous injection. Clinical signs of disease (onset of limb paralysis) were monitored and recorded by our veterinarian. Treatment continued until mice succumbed or the disease progression reached at least one of four defined endpoints: rapid body weight loss of >20%, multiple (or single large) tumor masses, anorexia, or failure to drink. Overall survival was analyzed by the Kaplan-Meier method.

### Heterotopic AML animal model

For establishing the xenograft animal model, 6 × 10⁶ Molm-13 cells were subcutaneously injected into the flank region of 7-week-old female B-NDG mice (NOD-Prkdcscid IL2rgtm1/Bcgen; Biocytogen, Beijing, China), with one dose per mouse.

Tumor volumes (V) were calculated by caliper measurement of the length (L) and width (W) of the tumors using the formula: Tumor volume = 0.5 × L × W^2^. When the tumor volume reached 400 mm^3^, the mice were randomly assigned to four treatment groups.

Mice received either 15 mg/kg cytarabine daily by intratumorally injection or 40 mg/kg STA-9090 by tail vein injection on days 1, 4, 6 of every 7 days, or both drugs. At 2-day intervals, tumor volumes and mouse body weights were assessed and recorded to monitor tumor growth and animal health status. When the tumor length reached 20 mm, the mice were humanely sacrificed, and tumors were dissected out for further H&E and IHC staining analysis.

### Cell co-culture system

Spleens were harvested from humanely sacrificed c57 mice and rinsed with 75% reagent alcohol. The tissue was mechanically dissociated by mincing and pressing it through a cell strainer using a syringe plunger. The strainer was rinsed with PBS, and the cell suspension was collected into a 15 mL conical tube. Cells were sedimented via centrifugation (1500 rpm, 5 min), then resuspended in 3–5 mL of RBC Lysis Buffer and incubated for 5–10 min at room temperature. Subsequently, the resulting murine splenocytes were washed with PBS to remove lysis buffer and cellular debris.

HeLa cells were plated in 12-well plates at a density of 2 × 10⁵ cells per well, followed by overnight incubation to allow cell adherence, then add with resuspended murine splenocytes (3 × 10^8^ cells/well). Cell co-culture with drug treatment for 48 h, murine splenocytes were separated from the culture system, and HeLa cells were rinsed with PBS to prepare them for subsequent experimental analyses.

### Kaplan–Meier survival analysis

Survival analysis was conducted using survminer package in R (Version 3.6.1). Level 3 FPKM RNAseq data and associated clinical information of BRCA, LGG and THYM were downloaded from UCSC Xnea. For each of the above tumor types, the samples were sorted in descending order according to the expression level of SAMHD1, and overall survival analysis was performed for the first (High expression group) and last quartile (Low expression group) of the samples.

### Ex vivo natural killer cell cytotoxicity assay

Natural killer (NK) cells were purified from umbilical cord blood obtained from Zhejiang Province Umbilical Cord Blood Hematopoietic Stem Cell Bank. Isolation began with the dilution of the umbilical cord blood, followed by Ficoll density gradient centrifugation to isolate the PBMCs. Subsequently, magnetic-activated cell sorting (MACS) using a specific NK cell isolation kit (Miltenyi, catalog number: 130-092-657) was employed to selectively enrich for NK cells based on CD56 expression. The enriched NK cell population was further purified through fluorescence-activated cell sorting (FACS) with targeted antibodies. Finally, the purity and functionality of the isolated NK cells were validated via flow cytometry and functional assays.

To determine the ability of NK cells to kill tumor cells in vitro, an equal number of HeLa cells were seeded into 12-well plates one day prior to the experiment. The following day, the cells were transfected with either a SAMHD1 expression vector or an empty plasmid control. Twelve hours post-transfection, HSP90 inhibitor was added at a specific concentration. After an 18 h drug treatment, co-cultivation was initiated at an effector-to-target (E:T) ratio of 10:1 (NK cells to Hela cells). Cell death was monitored continuously, and unbiased photomicrographs were captured under a light microscope. Viable cell counting was performed on cells from parallel wells to evaluate cytotoxicity.

### Statistical analysis

All statistical analyses were conducted using GraphPad Prism 6 software. Data are expressed as the mean ± standard deviation (s.d.). Group comparisons were analyzed via an unpaired two-tailed Student’s t-test, where *p*-values < 0.05 were defined as statistically significant. Significance levels are indicated as follows: ns (not significant); *p < 0.05; **p < 0.01; ***p < 0.001; ****p < 0.0001.

## Supplementary information

Supplementary Materials

Supplementary Table 2

## Data Availability

The mass spectrometry proteomics data were submitted to the ProteomeXchange Consortium (https://proteomecentral.proteomexchange.org) through the iProX partner repository, with the dataset identifier PXD070214.^72,73^ Other original data are available from the corresponding author, Prof. Xiaofang Yu (xfyu1@zju.edu.cn), upon reasonable request.
