# Supplementary material for: Selective depletion of tumor-associated SAMHD1 enhances chemotherapeutic efficacy and antitumor immune responses
Source: Signal Transduct Target Ther. 2025 Dec 15;10:406. doi: 10.1038/s41392-025-02523-1 (PMC12703001; doi:10.1038/s41392-025-02523-1)
Supplement: Supplementary file 1 — Supplementary Materials [file 41392_2025_2523_MOESM1_ESM.docx]

Supplementary Materials for

Selective depletion of tumor-associated SAMHD1 enhances chemotherapeutic efficacy and antitumor immune responses

Jing Sun1†, Wenwen Zheng1†, Zheng-Guo Zhang1†, Hongkun Zhou3†, Songdi Wang1†, Dingbo Huang1, Xiao-Yi Hu1, Qing-Feng Yu5, Zhao-Xing Wu5, Yi-Fei Shi1, Runxin Ye1, Fengyan Xia1, Wangwei Li8, Shurui Lyu1, Yu Huang6, Xu-Zhao Zhang5, Fei Xu5, Ke Zhao4, Jie Yang3, Juan Du4, Jiaming Su1, Yajuan Rui1, Rongzhen Xu5, Wei-Ming Yang2, Li Cang1, Jia ling Xu1, Ruiyu Zhu7,8, Xiaoguang Wang3*, Wei Wei7* and Xiao-Fang Yu1*

Correspondence to: Xiao-Fang Yu (Email: xfyu1@zju.edu.cn)

**This PDF file includes:**

Materials and Methods

Figures. S1 to S8

Tables S1 and S3

Materials and Methods

RNA silencing

The shRNA oligos are as follows:

sh-HSP90:

F:

5’CCGGGATCAGACAGAGTACCTAGCTCGACCTAGGTACTCTGTTCTGATCTTTTTG3’

R:

5’AATTCAAAAAGATCAGACAGAGTACCTAGCTCGAGCTAGGTACTCTGTTCTGATC3’

sh-SAMHD1:

F:

5’CCGGGCGGACGATTATATTGAAATACTCGACTATTTCAATATAATCGTCCGCTTTTTG3’.

R:

5’AATTCAAAAAGCGGACGATTATATTGAAATACTCGACTATTTCAATATAATCGTCCGC3’


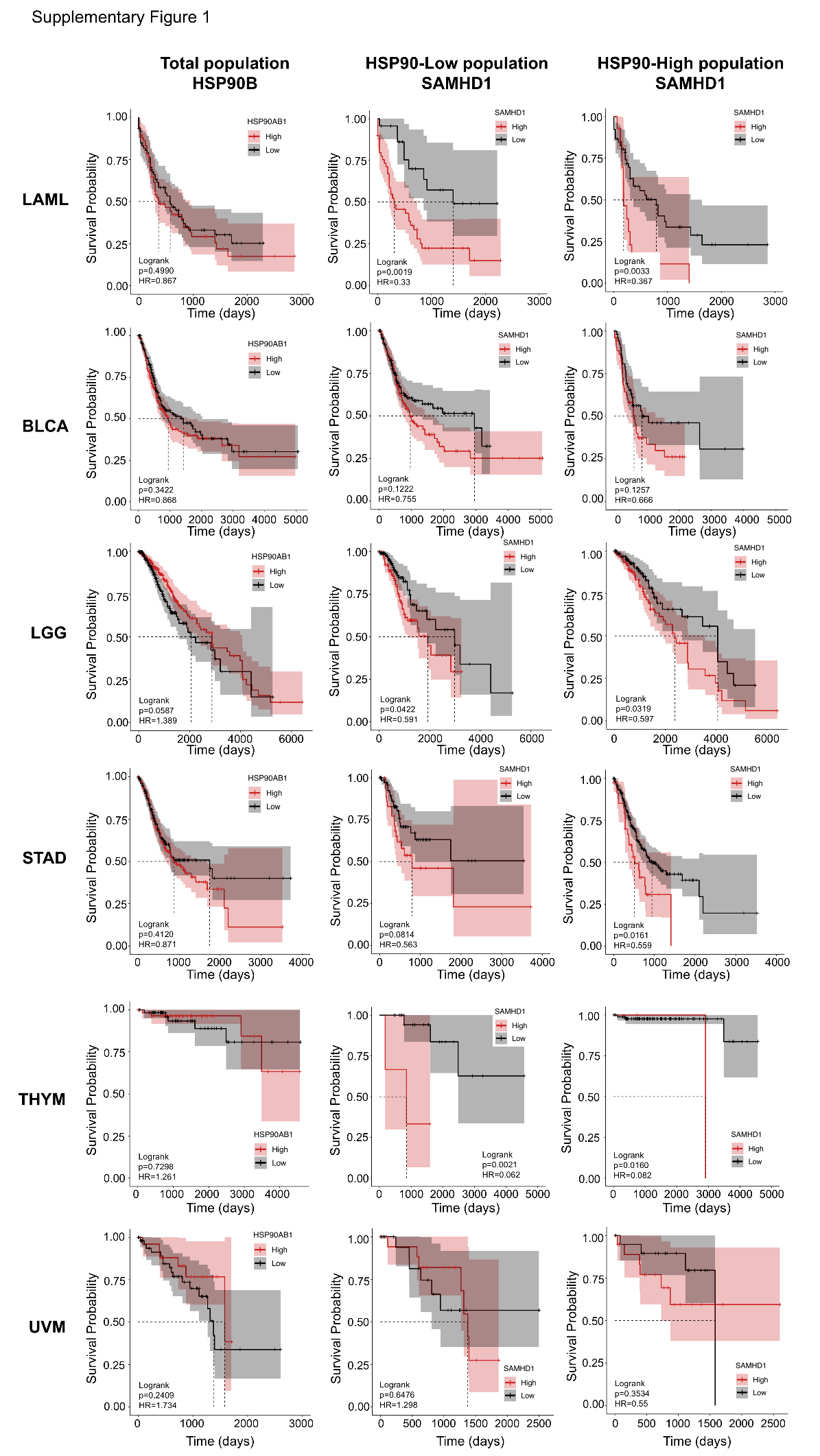
Figure. S1.

**Supplementary Figure 1 Kaplan–Meier survival analysis of cancer patients grouping by HSP90 RNA expression.** LAML, acute myeloid leukemia, BRCA, breast invasive carcinoma, LGG, brain lower-grade glioma, STAD, stomach adenocarcinoma, THYM, thymoma, UVM, uveal melanoma. Hazard ratio (HR) and p values were indicated on the plots.

Figure. S2.


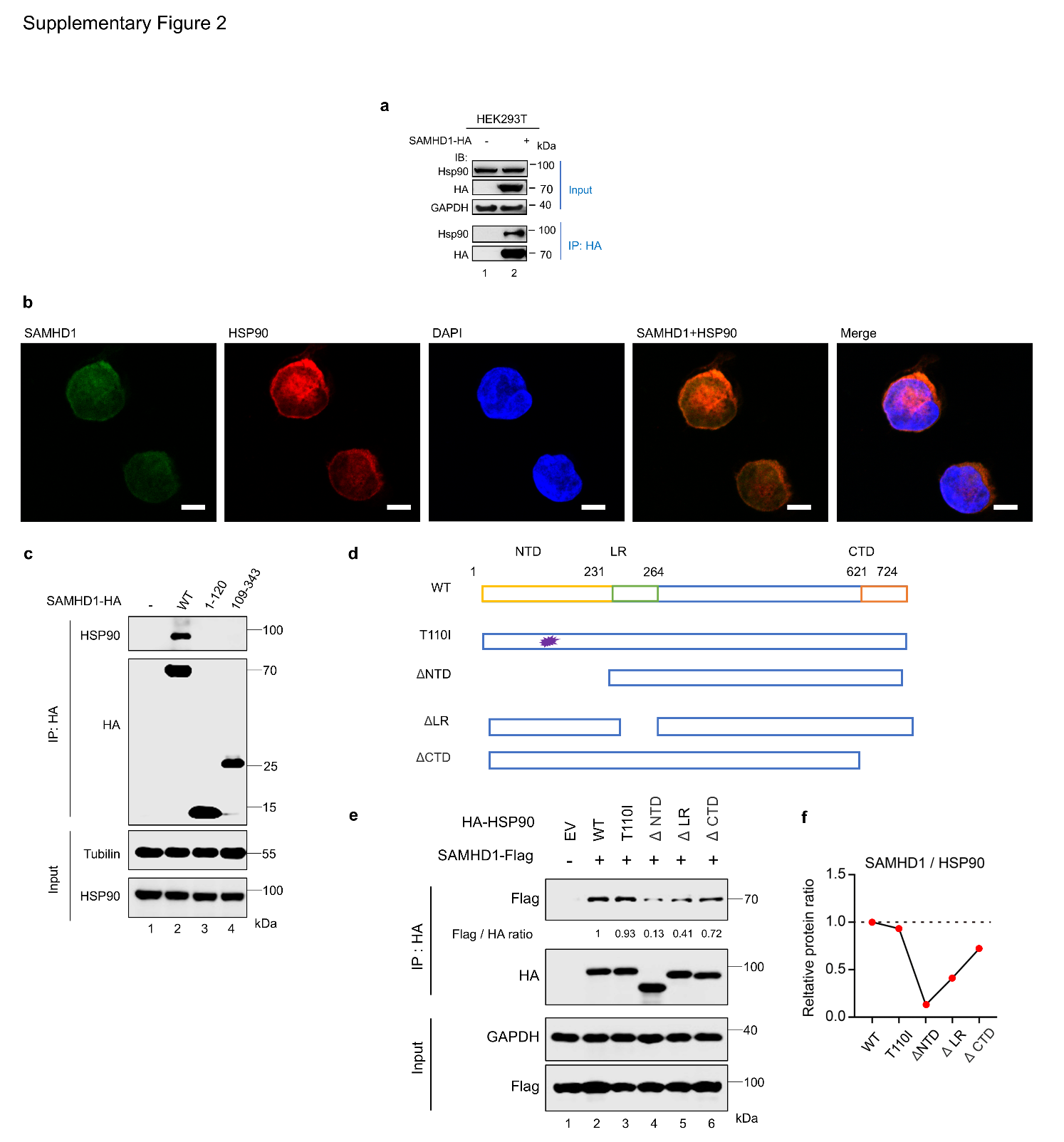
 **Supplementary Figure 2 Confirmation of endogenous SAMHD1 and HSP90 interaction. (a)** Co-IP assay confirming the SAMHD1–HSP90 interaction in HEK293T cells. Cells were transfected with the expression vector for HA-tagged SAMHD1, immunoprecipitated with the anti-HA beads, and analyzed by SDS-PAGE/Western blot with anti-HSP90 antibody (n = 3). **(b)** Detection of intracellular co-localization of SAMHD1 and HSP90 proteins by immunofluorescent staining. Subcellular co-localization of SAMHD1 and HSP90 was observed in the nuclei of Molm-13 cells. DAPI staining was used to visualize the nuclei (n=2). Scale bar, 10μm. **(c)** Co-IP assay demonstrating that the SAMHD1 amino acid fragment 1-343 is not required for mediating the interaction with HSP90. The designated HA-tagged SAMHD1 constructs were transfected into HEK293T cells, followed by co-IP performed with HA beads. Precipitated proteins were detected by Western blot analysis using anti-HSP90 antibody. **(d)** Schematic diagram of the HSP90 domain architecture and the corresponding co-IP experimental design used in (c). **(e)** Co-IP demonstrating that the NTD, LR, and CTD domains of HSP90 all contribute to the interaction with SAMHD1. Flag-tagged wild-type SAMHD1 and designated HA-tagged HSP90 truncation mutants (as shown in panel a) were co-transfected into HEK293T cells. The complexes were immunoprecipitated using anti-HA beads, and precipitated proteins were detected by Western blot analysis using anti-Flag antibody. **(f)** Densitometric analysis of co-immunoprecipitated Flag-SAMHD1 levels relative to precipitated HA-HSP90 protein levels.

Figure. S3.


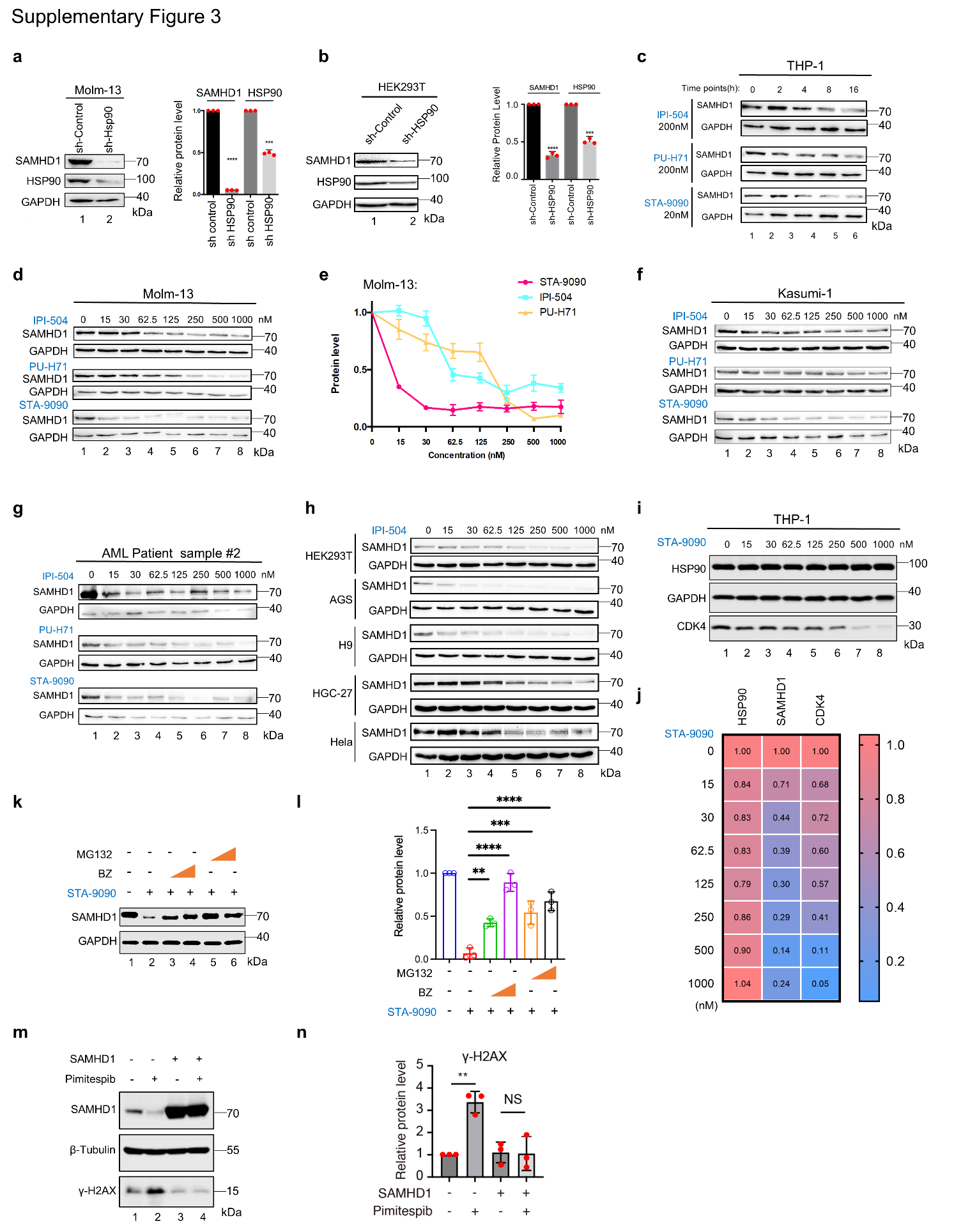
 **Supplementary Figure 3 HSP90 inhibition leads to SAMHD1 depletion in tumor cells.** **(a-b)** Western blot and quantification showing that shRNA-mediated knockdown of HSP90 in Molm-13 and HEK293T cells resulted in a corresponding decrease in SAMHD1 protein abundance (n=3). Data are represented as mean±s.d. *p<0.05; **p<0.01; ***p<0.001; ****p<0.0001. **(c-f)** Time-course evaluation of SAMHD1 protein expression levels in **(c)** THP-1, **(d-e)** Molm-13 and **(f)** Kasumi-1 cells treated with 200 nM IPI-504, 200 nM PU-H71, or 20 nM STA-9090. At the specified time points, cells were harvested and then subjected to Western blot analysis. **(g)**Western-blot analysis of primary blast from AML patient #2 treated with IPI-504, PU-H71, and STA-9090, respectively. **(h)** Western blot analysis of SAMHD1 protein expression in the indicated cell lines after 18-hour treatment with 200nM IPI-504. **(i-j)** Time- and dose-dependent analysis of HSP90 and CDK4 protein expression in THP-1 cells treated with indicated dose of STA-9090 for18 hours. **(j)** Heatmap showing the corresponding relative protein quantification of HSP90, SAMHD1 and CDK4 levels from the Western blot. **(k-l)** THP-1 cells were treated with the HSP90 inhibitor STA-9090 (500 nM) in combination with either: **k)** the proteasome inhibitor MG132 (1 μM or 5 μM), or **l)** bortezomib (10 nM or 50 nM) for 14 hours. Cells were thereafter collected to conduct Western blot analysis. Protein band quantification was performed using ImageJ. **(m-n)** HSP90 inhibitor-mediated SAMHD1 degradation enhances DNA damage, which is rescued by SAMHD1 reconstitution. Immunoblot analysis of γ-H2AX and SAMHD1 protein levels in HEK293T cells treated with or without Pimitespib (500nM) and/or upon SAMHD1 overexpression (n = 3).


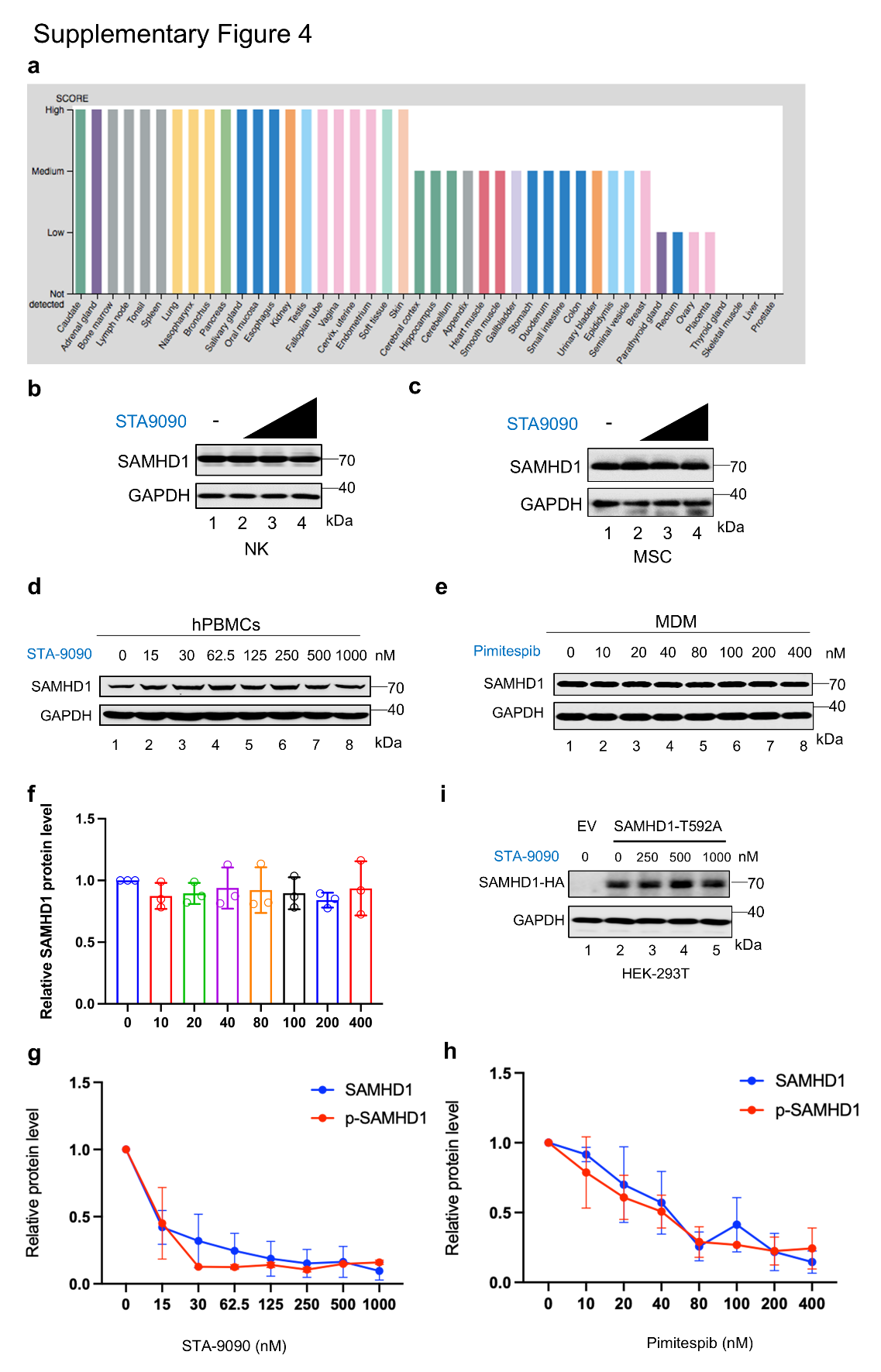
Figure. S4.

**Supplementary Figure 4: HSP90 inhibitors exhibited minimal effects on SAMHD1 expression in primary cells.** **(a)** Tissue-specific pattern of SAMHD1 protein expression derived from the Human Protein Atlas. **(b-d)** Western blot analysis of SAMHD1 protein expression in **(b)** NK cells, **(c)** MSCs (Mesenchymal stem cell), and **(d)** hPBMCs after 18h treatment with the indicated doses of STA-9090. GAPDH acted as a loading control for sample loading (n = 3). **(e-f)** CD14+ cells from healthy hPBMCs were isolated using CD14+ magnetic microbeads and treated with 10 ng/mL human GM-CSF for 7 days. Differentiated macrophages were treated with various doses of Pimitespib for 18 hours (n = 3). Protein band quantification was performed using ImageJ. **(g-h)** Line graphs illustrating phosphorylated SAMHD1 (Thr592) and total SAMHD1 protein levels in THP-1 cells following 18-hour exposure to specified doses of **(g)** STA-9090 and **(h)** Pimitespib. **(i)** Exogenous SAMHD1-T592A-HA was transfected into HEK-293T cells, followed by treatment with gradually increasing doses of STA-9090 for 18 hours. The level of HA-tagged SAMHD1-T592A in the cells was detected by Western blot, with GAPDH as the loading control.

Figure. S5.


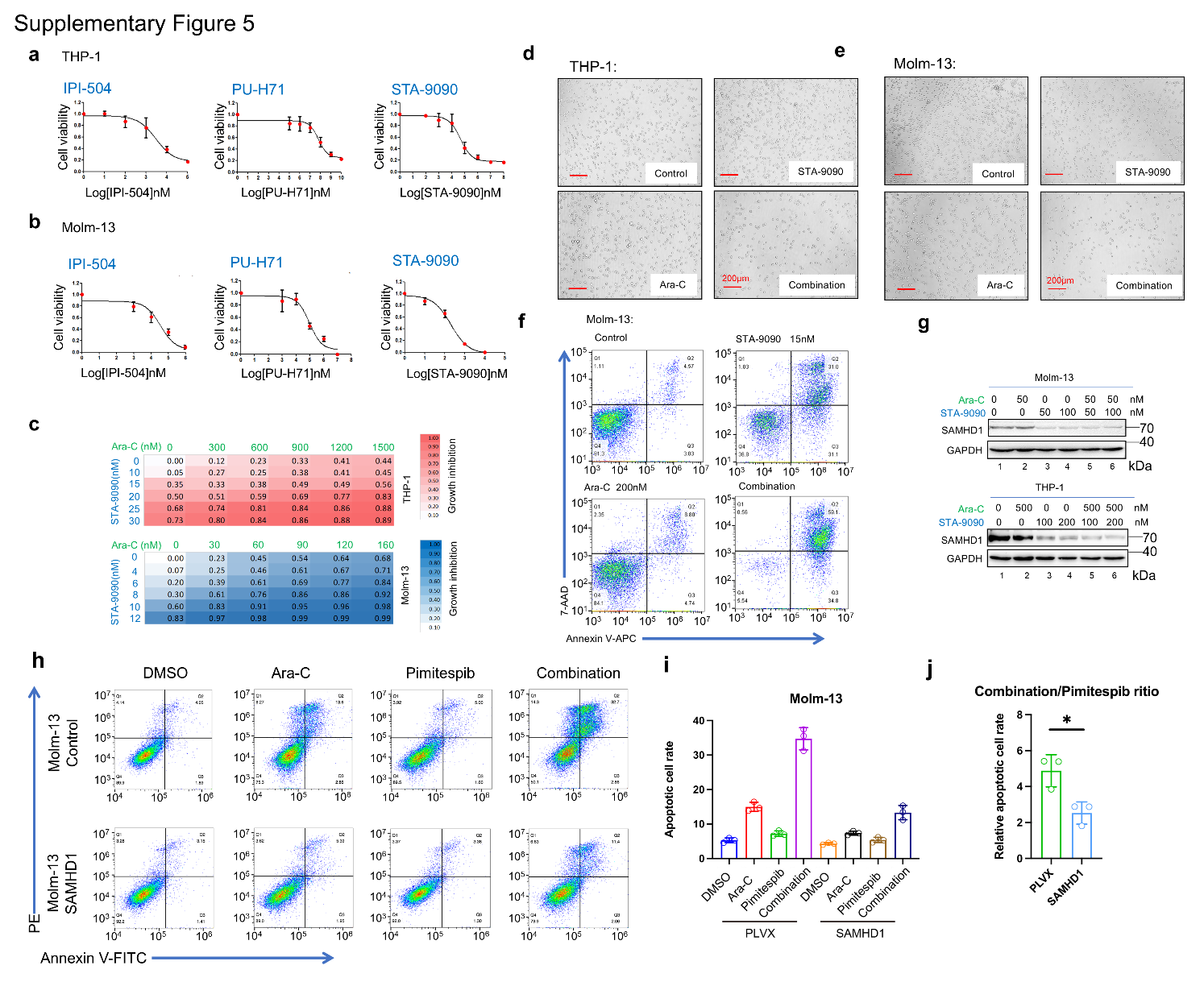
 **Supplementary Figure 5: HSP90 inhibitors and ara-C exert a synergistic effect in killing AML cells.** **(a-b)** Dose-response curves for THP-1 cells **(a)** and Molm-13 cells **(b)** treated with IPI-504, PU-H71 and STA-9090 for 72 hours. MTT assay was employed to determine cell viability (n = 3). Data are shown as mean± s.d. **(c)** Heatmap showing the growth inhibition rate by different dose of STA-9090 and ara-C combination in THP-1 and Molm-13. **(d-e)** Microscopic images of **(d)** THP-1 and **(e)** Molm-13 cells after STA-9090, ara-C and combined treatment. **(f)** Flow cytometry analysis quantifying apoptosis in Molm-13 induced by STA-9090, ara-C and combined treatment. **(g)** Western blot analysis of SAMHD1 protein after treatment with STA-9090, ara-C, or in combination in Molm-13 and THP-1 cells (n=3). **(h-j)** Flow cytometry analysis quantifying apoptosis in SAMHD1-overexpressing or control Molm-13 cells induced by STA-9090, ara-C and combined treatment.

Figure. S6.


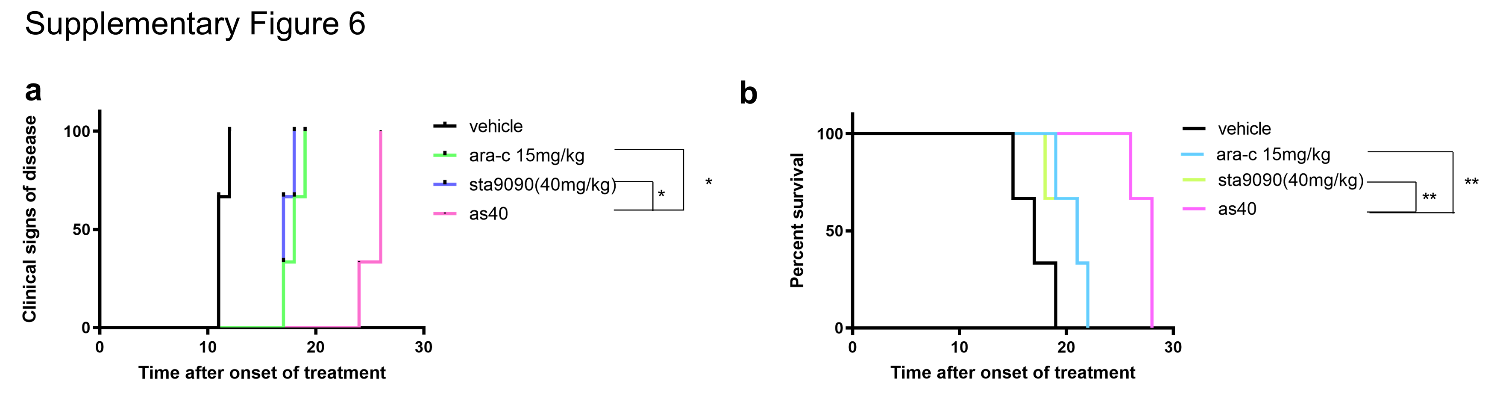
 **Supplementary Figure 6 Clinical signs of disease and Kaplan-Meier survival analysis in AML mouse models.** **(a-b)** The ara-C and STA-9090 single-treatment groups were started one week earlier than the combination group. **(a)** Clinical sign of disease progression among different groups, specifically the onset of hind limb paralysis. **(b)** Kaplan-Meier survival analysis for all treatment groups. The Mantel-Cox log-rank test was employed to determine survival differences.


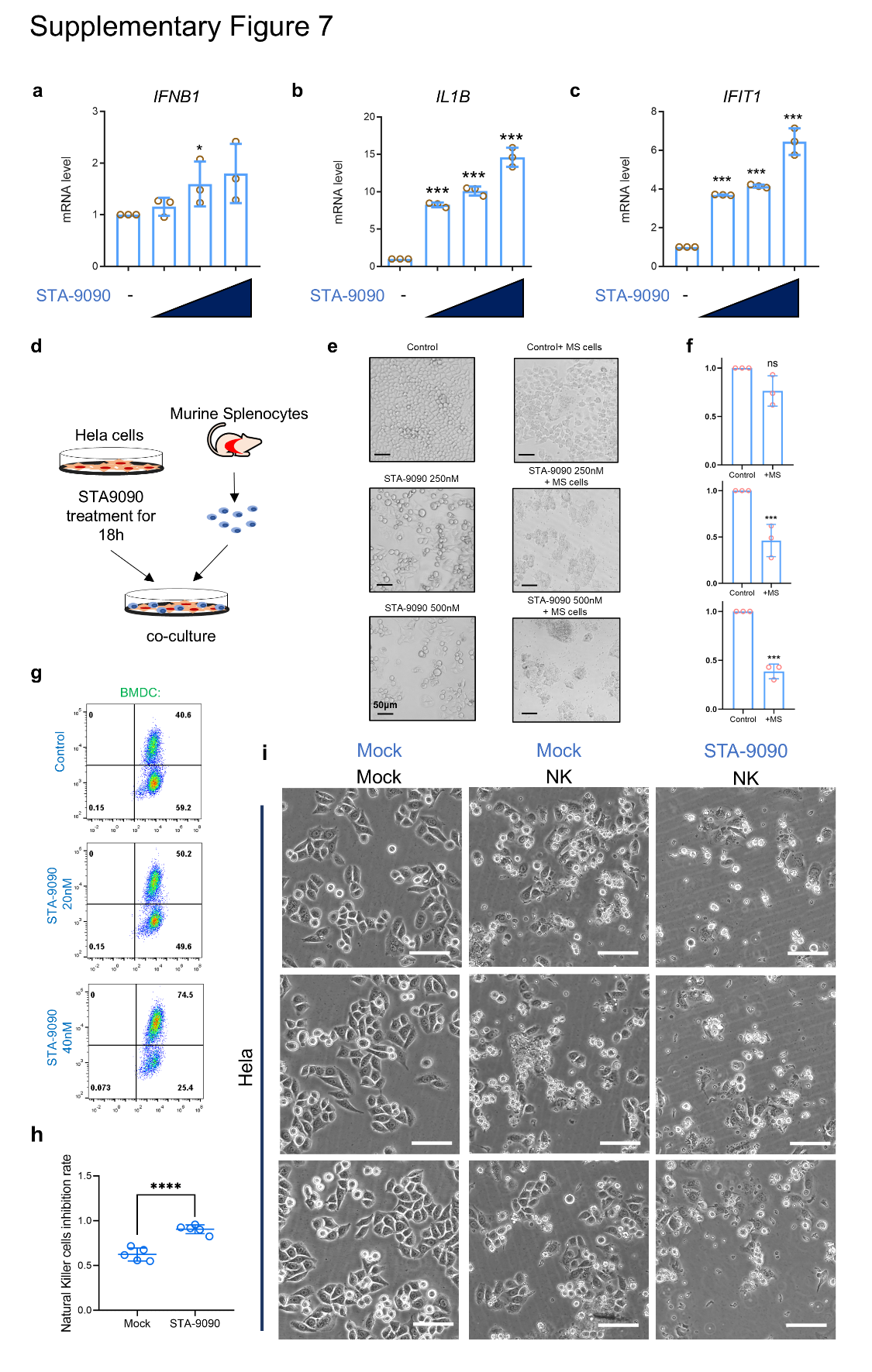
Figure. S7.

**Supplementary Figure 7 STA9090 triggers innate immune responses in tumor cells and enhances anti-tumor immunity.** **(a-c)** Quantitative RT-PCR analysis depicting increased expression of genes related to antiviral and inflammatory cytokines (*IFNB, IL1B, IFIT1*) in HeLa cells after 18 hours of treatment with designated concentrations of STA-9090 (n = 3). Data are presented as mean ± s.d. **(d)** Schematic model illustrating murine splenocytes and tumor cell co-culture system. **(e)** Light microscopy images of Hela cells treated with various doses of STA-9090 with or without murine splenocytes. **(f)** Trypan blue counting of viable cells from **(e)** three independent experiments. **(g)** STA-9090 treatment for 24h facilitates the maturation of murine dendritic cells (n=3). **(h-i)** Synergistic effects of STA-9090 and NK cell–mediated cytotoxicity. Typical phase-contrast micrographs illustrating morphological alterations in HeLa cells following 24-hour exposure to STA-9090 as monotherapy or combined with NK cells (E:T ratio = 10:1). Scale bar, 100 μm. (h) Quantitative assessment of NK-cell cytotoxic activity. Following 24 hours of co-culture, target cell viability was ascertained via live cell staining and counting. Data were normalized to the untreated control and shown as mean ± s.d. of three independent experiments.

Figure. S8.


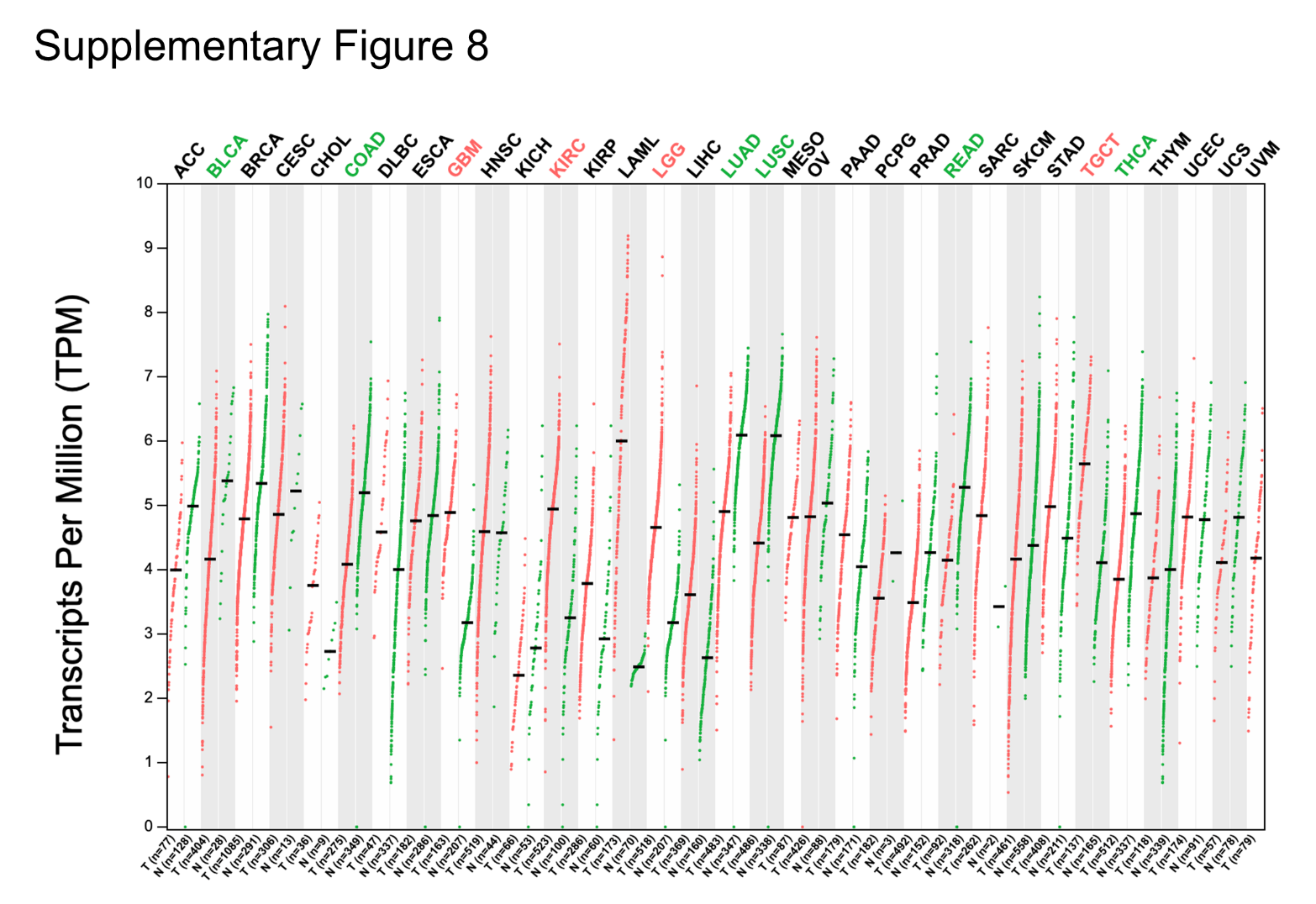
 **Supplementary Figure 8: SAMHD1 expression in samples of different tumors vs. matched normal.** Dot plot showing the pan-cancer gene expression analysis of SAMHD1 in tumor and paired normal tissues. Data was analyzed using GEPIA2 and The Cancer Genome Atlas (TCGA) database. The cancer type abbreviations are color-coded to indicate statistically significant differential expression. Red labels signify a significant upregulation of SAMHD1 in tumor tissues, while green labels signify a significant downregulation. T, Tumor; N, Normal.

Table S1.

| Drug Number | Chemical Abstracts Service Number (CAS No.) |
| --- | --- |
| 1 | 888216-25-9 |
| 2 | 868540-17-4 |
| 3 | 34157-83-0 |
| 4 | 61825-94-3 |
| 5 | 915019-65-7 |
| 6 | 97858-29-8 |
| 7 | 179324-69-7 |
| 8 | 158442-41-2 |
| 9 | 56238-63-2 |
| 10 | 21679-14-1 |
| 11 | 58957-92-9 |
| 12 | 27164-46-1 |
| 13 | 59865-13-3 |
| 14 | 127-07-1 |
| 15 | 118-42-3 |
| 16 | 162808-62-0 |
| 17 | 97682-44-5 |
| 18 | 51-21-8 |
| 19 | 97867-33-9 |
| 20 | 121032-29-9 |
| 21 | GC376 sodium |
| 22 | 154-17-6 |
| 23 | 53123-88-9 |
| 24 | 114977-28-5 |
| 25 | 3543-75-7 |
| 26 | 159989-65-8 |
| 27 | 151096-09-2 |
| 28 | 1426138-42-2 |
| 29 | 123318-82-1 |
| 30 | 50-18-0 |
| 31 | 59277-89-3 |
| 32 | 210344-98-2 |
| 33 | 155213-67-5 |
| 34 | 74578-69-1 |
| 35 | 127779-20-8 |
| 36 | 210345-00-9 |
| 37 | 159351-69-6 |
| 38 | 33069-62-4 |
| 39 | 86386-73-4 |
| 40 | 2138299-29-1 |
| 41 | 39831-55-5 |
| 42 | 32986-56-4 |
| 43 | 183321-74-6 |
| 44 | 66-27-3 |
| 45 | 100986-85-4 |
| 46 | 60-33-3 |
| 47 | 15663-27-1 |
| 48 | 54-05-7 |
| 49 | 1492-18-8 |
| 50 | 257933-82-7 |
| 51 | 1135695-98-5 |
| 52 | 66-81-9 |
| 53 | 70-00-8 |
| 54 | 147-94-4 |
| 55 | 210344-95-9 |
| 56 | 5536-17-4 |
| 57 | 210344-92-6 |
| 58 | 196309-76-9 |
| 59 | 273404-37-8 |
| 60 | 1187594-09-7 |
| 61 | 846557-71-9 |
| 62 | 103476-89-7 |
| 63 | 220620-09-7 |
| 64 | 1616493-44-7 |
| 65 | 35807-85-3 |
| 66 | 19542-67-7 |
| 67 | 117-39-5 |
| 68 | 58-32-2 |
| 69 | 178603-78-6 |
| 70 | 377090-84-1 |
| 71 | 915019-65-7 |
| 72 | 134381-21-8 |
| 73 | 61422-45-5 |
| 74 | 75330-75-5 |
| 75 | 1397-89-3 |
| 76 | 18323-44-9 |
| 77 | 1009298-09-2 |
| 78 | 192725-17-0 |
| 79 | 62893-20-3 |
| 80 | 97867-33-9 |
| 81 | 85622-93-1 |
| 82 | 403848-57-7 |
| 83 | 100986-85-4 |
| 84 | 302962-49-8 |
| 85 | 31430-18-9 |
| 86 | 92636-39-0 |
| 87 | 223537-30-2 |
| 88 | 923564-51-6 |
| 89 | 847499-27-8 |
| 90 | 529-44-2 |
| 91 | 406209-26-5 |
| 92 | 23214-92-8 |
| 93 | 1028385-32-1 |
| 94 | 198904-31-3 |
| 95 | 571190-30-2 |
| 96 | 151767-02-1 |
| 97 | 134036-52-5 |
| 98 | 749886-87-1 |
| 99 | 60940-34-3 |
| 100 | 59703-84-3 |
| 101 | 923604-59-5 |
| 102 | 64485-93-4 |
| 103 | 88899-55-2 |
| 104 | 30827-99-7 |
| 105 | 83-46-5 |
| 106 | 130370-60-4 |
| 107 | 495-12-9 |
| 108 | 33419-42-0 |
| 109 | 1809249-37-3 |
| 110 | 26833-87-4 |
| 111 | 50-07-7 |
| 112 | 1231929-97-7 |
| 113 | 7689-03-4 |
| 114 | 50-89-5 |
| 115 | 58970-76-6 |
| 116 | 95058-81-4 |
| 117 | 197855-65-5 |
| 118 | 199986-75-9 |
| 119 | 41575-94-4 |
| 120 | 66701-25-5 |
| 121 | 133407-82-6 |
| 122 | 134448-10-5 |

Table S3.

| **Characteristics of primary AML samples** | | | | | | | | | |
| --- | --- | --- | --- | --- | --- | --- | --- | --- | --- |
| **ID** | **Sample type** | **age at diagnosis** | **gender** | **complete remission** | **WHO classification** | **cytogenetics** | **FLT3** | **treatment** | **outcome** |
| **P1** | **diagnosis** | **64** | **male** | **no** | **M2a** | **normal** | **negative** | **decitabine, cytarabine, homoharringtonine, idarubicin** | **not alive** |
| **P2** | **diagnosis** | **62** | **male** | **no** | **M2a** | **DNMT3a: c.2645G>A** | **negative** | **IA (cytarabine+ idarubicin), methotrexatum** | **receiving treatment** |
